# EMF1 and PRC2 Cooperate to Repress Key Regulators of Arabidopsis Development

**DOI:** 10.1371/journal.pgen.1002512

**Published:** 2012-03-22

**Authors:** Sang Yeol Kim, Jungeun Lee, Leor Eshed-Williams, Daniel Zilberman, Z. Renee Sung

**Affiliations:** Department of Plant and Microbial Biology, University of California Berkeley, Berkeley, California, United States of America; The University of North Carolina at Chapel Hill, United States of America

## Abstract

*EMBRYONIC FLOWER1* (*EMF1*) is a plant-specific gene crucial to Arabidopsis vegetative development. Loss of function mutants in the *EMF1* gene mimic the phenotype caused by mutations in Polycomb Group protein (PcG) genes, which encode epigenetic repressors that regulate many aspects of eukaryotic development. In Arabidopsis, Polycomb Repressor Complex 2 (PRC2), made of PcG proteins, catalyzes trimethylation of lysine 27 on histone H3 (H3K27me3) and PRC1-like proteins catalyze H2AK119 ubiquitination. Despite functional similarity to PcG proteins, EMF1 lacks sequence homology with known PcG proteins; thus, its role in the PcG mechanism is unclear. To study the EMF1 functions and its mechanism of action, we performed genome-wide mapping of EMF1 binding and H3K27me3 modification sites in Arabidopsis seedlings. The EMF1 binding pattern is similar to that of H3K27me3 modification on the chromosomal and genic level. ChIPOTLe peak finding and clustering analyses both show that the highly trimethylated genes also have high enrichment levels of EMF1 binding, termed EMF1_K27 genes. EMF1 interacts with regulatory genes, which are silenced to allow vegetative growth, and with genes specifying cell fates during growth and differentiation. H3K27me3 marks not only these genes but also some genes that are involved in endosperm development and maternal effects. Transcriptome analysis, coupled with the H3K27me3 pattern, of EMF1_K27 genes in *emf1* and PRC2 mutants showed that EMF1 represses gene activities via diverse mechanisms and plays a novel role in the PcG mechanism.

## Author Summary

Polycomb group (PcG) proteins are epigenetic repressors maintaining developmental states in eukaryotic organisms. Plant PcG proteins are expected to be general epigenetic repressors; however, their overall impact on growth and differentiation and their mechanism of repression are still unclear. Here we identified several thousand target genes of the EMBRYONIC FLOWER 1 (EMF1) protein, which shares no sequence homology with known PcG proteins. EMF1 regulates developmental phase transitions as well as specifies cell fates during vegetative development. Trimethylation of histone 3 lysine 27 (H3K27me3) and ubiqutination of lysine 119 of histone H2A are carried out by different PcG protein complexes. EMF1 is required for both histone modifications on genes specifying stem cell fate in plants, thus revealing a novel role of EMF1 in linking the PcG protein complexes. Our results have important implications for the evolution of PcG regulatory mechanisms.

## Introduction

Polycomb group (PcG) proteins are epigenetic repressors implicated in various developmental and cellular processes [1], [2]. PcG proteins function in multi-subunit protein complexes: Polycomb Repressor Complex 1 (PRC1) and PRC2 [3], the core components of which are conserved from Drosophila to humans. PRC2 marks the target gene by trimethylating histone H3 at lysine 27 (H3K27me3) through the E(z) SET domain [4], [5], [6], [7], [8]. PRC1, which binds the H3K27me3 methyl marks and docks on nucleosomes modified by PRC2, inhibits transcription and blocks remodeling of the target nucleosomes, resulting in gene silencing [9], [10], [11]. Genome-wide studies confirmed co-localization of PRC1 and PRC2 on target genes. However, there are also genomic sites bound by one, but not the other, PRC [12] and transcriptional networks differentially regulated by PRC1 and PRC2 [13]. PcG action is counteracted by Trithorax Group (trxG) protein complexes [14]. Together, PcG and trxG complexes maintain repressive and active states of chromatin, respectively [14].

Protein-protein interaction and gel filtration studies have identified three Arabidopsis PRC2-like complexes [15], [16], [17]. Two components, FERTILIZATION INDEPENDENT ENDOSPERM (FIE) [18], and MULTICOPY SUPPRESSOR OF IRA1 (MSI1) [19], are present in all three putative PRC2s [17]. Small gene families of homologs of Drosophila Su(z)12, i.e., EMBRYONIC FLOWER2 (EMF2) [20], FERTILIZATION INDEPENDENT SEED2 (FIS2) and VERNALIZATION2 (VRN2) [21], and of E(z), i.e., MEDEA (MEA) [22], CURLY LEAF (CLF) [23], and SWINGER (SWN) [15], generate variation in Arabidopsis complex composition for targeted PRC2 regulation of multiple pathways.

The EMF2/FIS2/VRN2 homologs have diverse, and sometimes redundant, roles [24], [25], [26]. The VRN2-containing PRC2, VRN2-PRC2, is required for vernalization-induced flowering through the repression of *FLOWERING LOCUS C* (*FLC*) [21]. Impairments in FIS2-PRC2 function cause endosperm over-proliferation and seed abortion [26]. Impairments in the EMF2-PRC2 do not affect seed development, but the plants have a shortened vegetative phase or skip it altogether [18], [20], [23], [27]. Hence, EMF2-PRC2 is considered responsible for vegetative development.

*EMF1*, another Arabidopsis gene required for vegetative development, encodes a plant-specific protein containing sequence motifs found in transcriptional regulators [28]. EMF1 mutant plants and plants impaired in components of EMF2-PRC2 have similar phenotypes. Weak *emf1* mutants are *emf2*-like, while strong *emf1* mutants have a more severe phenotype than *emf2* and the transgenic lines impaired in *FIE* [18], [27], [29], [30]. Tissue-specific removal of EMF1 activity from leaf primordia allows vegetative growth, but leads to early flowering plants with curly leaves similar to *clf* mutants [31]. The early flowering phenotype of plants impaired in *EMF1* or EMF2-PRC2 components was attributed to the ectopic expression of flower organ identity or flower *MADS box* genes such as *AGAMOUS* (*AG*), *APETALA1* (*AP1*), *AP3* and *PISTILATA* (*PI*) [32], . However, these plants have pleiotropic phenotypes and the expression of many genes other than the flower *MADS box* genes is affected [33], [34], [35]. This suggests that EMF1 and EMF2-PRC2 regulate additional developmental processes.

EMF1 interacts with *AG*, *PI*, and *AP3* chromatin and displays characteristics similar to the Drosophila PRC1 component, Posterior sex combs (Psc) [36]. It is also required for Arabidopsis RING-finger protein-mediated Histone 2A lysine 119 (H2AK119) ubiquitination [37]. Mammalian PRC1 contains the RING-finger proteins from an E3 ubiquitin ligase complex that monoubiquitinates H2AK119 [38]. Functional characterization of Arabidopsis RING-finger proteins provided biochemical, molecular, and biological evidence that they have a PRC1 role in maintaining differentiated cell fates [37], [39], [40]. Another Arabidopsis PRC1-like component, the LIKE HETEROCHROMATIN PROTEIN1 (LHP1), recognizes H3K27me3 and interacts with many H3K27 trimethylated target genes [41], [42]. The RING-finger proteins interact with both LHP1 and EMF1; and EMF1 is required for the H2AK119 ubiquitination activity of the RING-finger proteins [37]. However, EMF1 also interacts with the PRC2 component, MSI1, *in vitro* [36] as well as with multiple other proteins [43]. The role of EMF1 in the PcG mechanism remains unclear.

To better understand the full impact of EMF1 on Arabidopsis growth and development and the mechanisms of EMF1-mediated gene repression, we performed genome-wide mapping of EMF1 binding and analyzed the H3K27me3 and expression patterns of EMF1 target genes in *emf1* and PRC2 mutants. Our results demonstrate direct epigenetic regulation of key genes controlling developmental programs and specifying cell differentiation processes via their interaction with EMF1. Based on the requirement of EMF1 for H3K27me3 and H2AK119 ubiquitination on different target genes, we discuss the roles of EMF1 in the PcG mechanism and propose a novel role for EMF1– acting as a linker between the two PcG complexes for genes that depend on EMF1 for both histone modifications.

## Results

### Genome-wide EMF1 binding map in Arabidopsis seedlings

We have previously shown that EMF1 regulates the flower *MADS box* genes *AG*, *AP3*, and *PI* via direct interaction with their chromatin [34], [36]. The large number of mis-regulated genes in *emf1* mutants [33], [34] indicates that EMF1 regulates many other genes directly or indirectly. To identify all EMF1 target genes in Arabidopsis seedlings, we performed Chromatin Immunoprecipitation (ChIP) followed by microarray analysis (ChIP-chip), using a transgenic Arabidopsis with a functional transgene – *EMF1* tagged with *3FLAG* and expressed under its own promoter (*EMF1::EMF1-3FLAG*) that can rescue *emf1* mutants [36]. A high-resolution genome-wide map of EMF1 binding sites in Arabidopsis seedlings was generated by affinity purifying 3FLAG tagged EMF1-bound chromatin and hybridizing the associated DNA to customized NimbleGen High Density 2 tiling microarrays (HD2, 2.1M array) representing the entire Arabidopsis genome of 28,244 genes without gaps.

**Figure 1:**
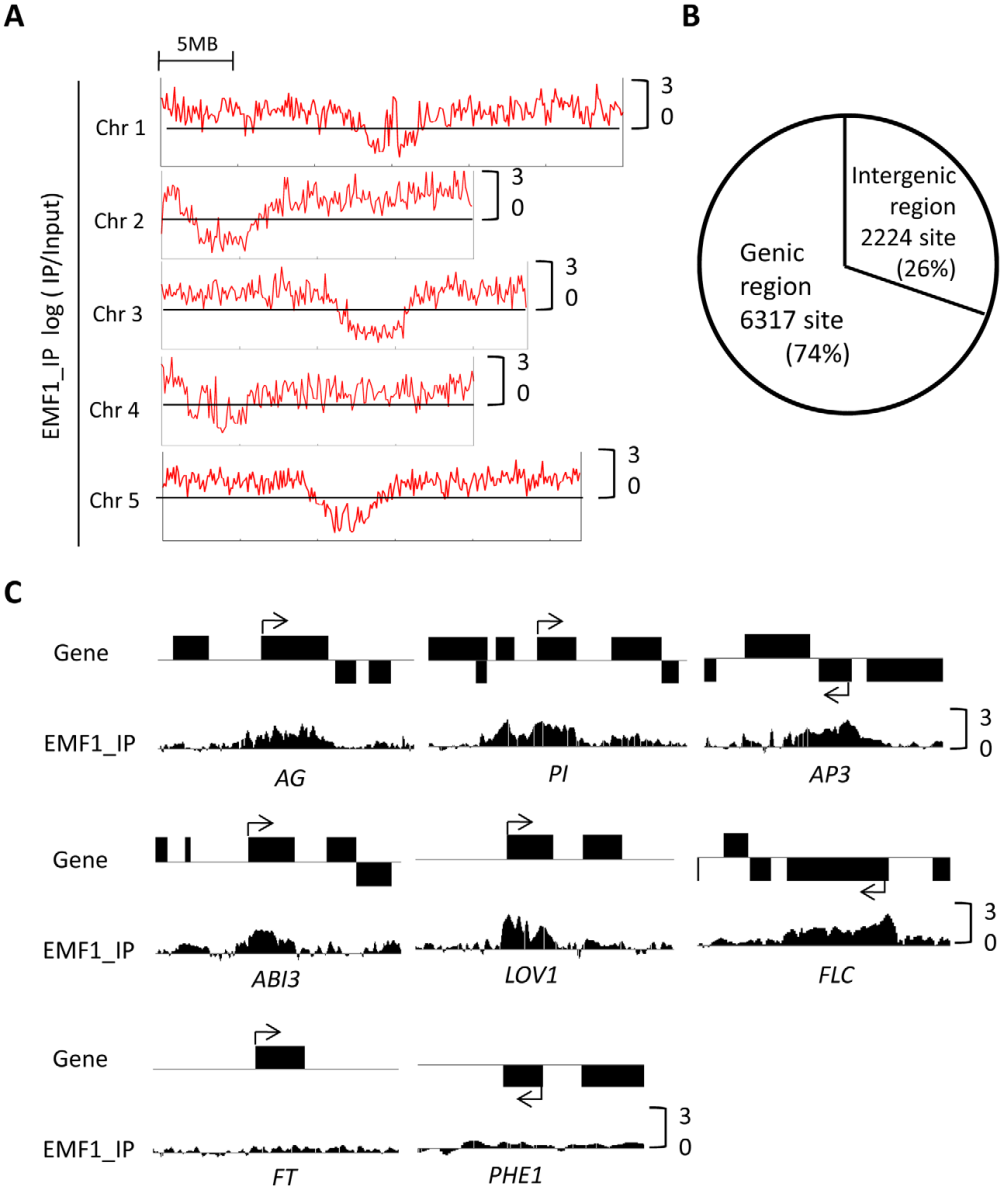
Genome-wide EMF1 binding map. (A) Chromosomal distribution of EMF1 binding sites. EMF1 binding regions per 100 kb on the 5 Arabidopsis chromosomes. Chr and 5MB represent chromosome and 5 megabase, respectively. Y-axis represents log_2_-ratio of the input signals for the immunoprecipitated DNA (IP/input). (B) EMF1 binding sites in genic and intergenic regions. (C) EMF1 binding pattern on Arabidopsis genes. Consistent with ChIP-PCR results [34], [36], *PI*, *AG*, *AP3*, *ABI3*, *LOV1*, and *FLC* chromatin is enriched with EMF1-3FLAG signal, but not *FT* and *PHE1*. Black box represents gene body and arrow indicates transcription start site (TSS) and transcriptional direction.

Utilizing the ChIPOTLe peak finding algorithm we identified 8,541 binding sites (p<10^−6^) distributed throughout all 5 chromosomes, enriched in the euchromatic regions and underrepresented in the pericentromeric region (Figure 1A; Figure S1A). 6,317 of the EMF1 binding sites are located in the transcribed region of the annotated sequences (−200 bp to the 3′ end) of 5,533 genes. The remaining sites are in intergenic regions (Figure 1B; Table S1). The 5,533 include *AG*, *AP3* and *PI* (Figure 1C), the known EMF1 target genes that are up-regulated in *emf1* mutants, as well as 7 other flower *MADS box* genes and *CRABS CLAW* (*CRC*) (Figure S1B). This is consistent with EMF1 repression of the flower organ program in Arabidopsis seedlings. Other EMF1 target genes identified by ChIP-PCR by Kim et al., [34], namely, *LONG VEGETATIVE1* (*LOV1*), *FLC*, and *ABSCISIC ACID INSENSITIVE3* (*ABI3*), are EMF1 binding genes in our study. As negative controls, *FLOWERING LOCUS T* (*FT*) and *PHERES1* (*PHE1*), which did not interact with EMF1 in ChIP-PCR experiments, are not enriched with EMF1 binding sites (Figure 1C). We confirmed the ChIP-chip results by ChIP-PCR on an additional 9 randomly selected genes with various enrichment level of EMF1 binding (Figure S2). Thus binding sites identified by ChIP-chip likely represent *in vivo* EMF1-target genes interaction.

### EMF1 binding correlates with H3K27me3, which depends on PRC2 and partially on EMF1

**Figure 2:**
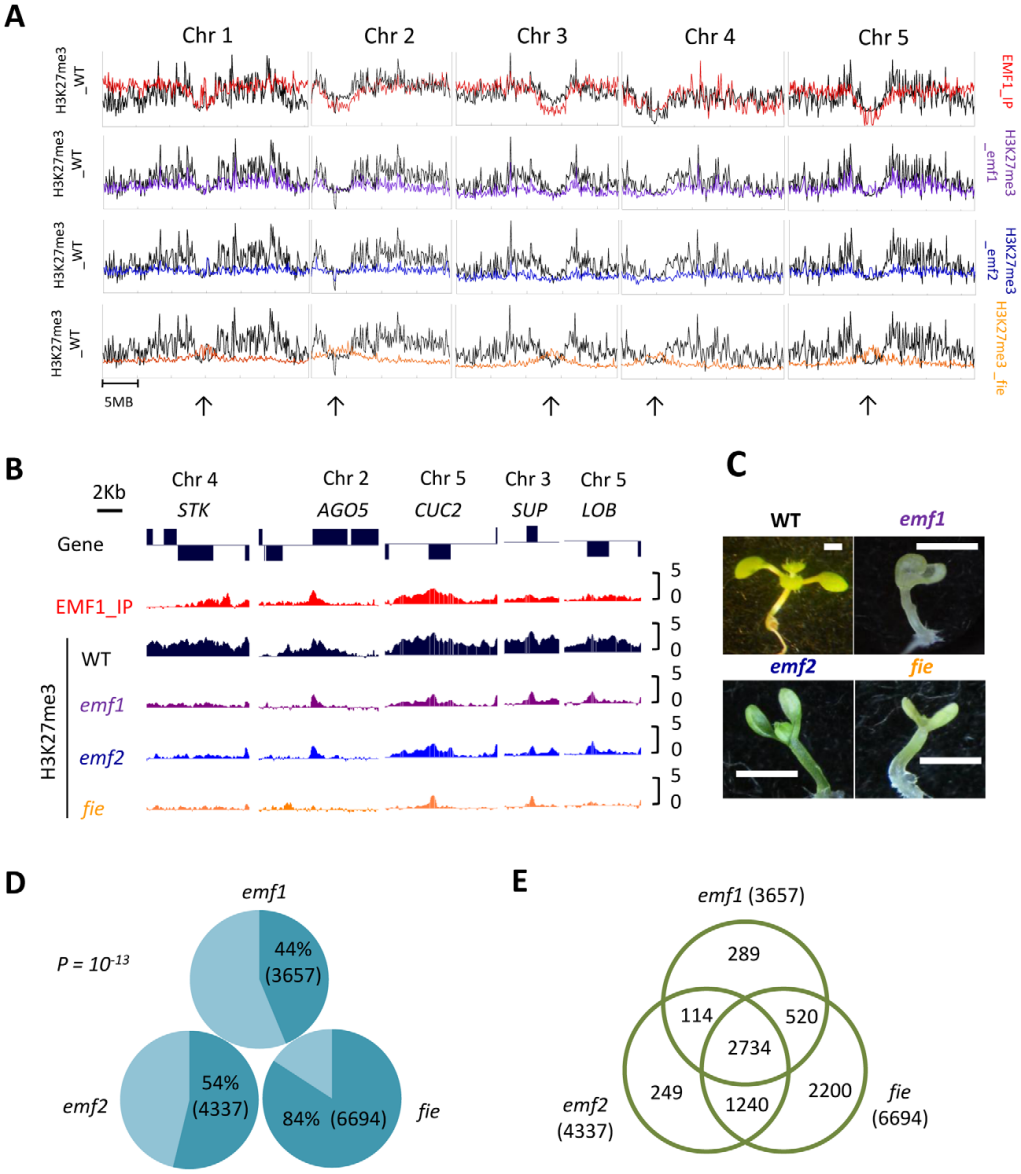
Chromosomal distribution of EMF1 binding sites and H3K27me3 modified regions in WT and 3 mutants. (A) Top panel shows the comparison of H3K27me3 marked (black) and EMF1 binding (red) regions per 100 kb in WT on 5 chromosomes. Lower three panels show comparison of H3K27me3 in WT and three mutants (purple: *emf1*; blue: *emf2*; orange: *fie*). Arrows point at pericentromeric locations. (B) H3K27me3 and EMF1 binding patterns (EMF1_IP) on individual genes in WT and 3 mutants. *STK*: *SEEDSTICK*, *AGO5*: *ARGONAUTE5*, *CUC2*: *CUP-SHAPED COTYLEDON2*, *SUP*: *SUPERMAN*, *LOB*: *LATERAL ORGAN BOUNDARIES*. (C) WT and mutants grown at short day condition for 15 days. (D) The percentage of 7,751 H3K27 trimethylated genes showing reduced methylation in mutants. 44%, 54% and 84% of the H3K27me3 marked genes in WT show reduced methylation in *emf1*, *emf2* and *fie* mutants, respectively. The p-value is 10^−13^. (E) Venn diagram showing number of genes with reduced methylation that overlaps.

Because of the functional similarity between EMF1 and PRC2, we compared the EMF1 binding pattern and the H3K27me3 modification profile across the whole Arabidopsis seedling genome. To minimize variability due to sample and microarray differences, we mapped EMF1 binding targets, determined the H3K27me3 profile, and measured mRNA levels (see below) with the same NimbleGen HD2 arrays. The ChIPOTle peak finding program identified 11,067 H3K27me3 enriched peaks (p<10^−35^), which correspond to 7,751 genes that showed 85% overlap with an earlier study (Table S2; [42]). As reported previously, H3K27me3 peaks tend to be broad, often covering the entire transcriptional unit (Figure 2A and 2B; Figure S1B), hence we used a very strict statistical cutoff for peak identification.

Globally, EMF1 binding and H3K27me3 modification profiles are well correlated (Figure 2A). Both are found throughout euchromatin regions and are underrepresented in the centromeres of all 5 chromosomes. At the genic level, the EMF1 binding pattern resembles the H3K27me3 profile, covering the transcription unit with the strongest signal around the transcriptional start site (TSS, Figure 1C). The EMF1 signal gradually declines towards the 3′ end in some genes and does not extend as far into the 3′ non coding region as H3K27me3 modification does, see, for example, *SEEDSTICK* (*STK*), *ARGONAUTE5* (*AGO5*), *AP1*, and *SEPALATA1* (*SEP1*) (Figure 2B; Figure S1B).

To better understand the relationship between EMF1 and PRC2, we mapped the H3K27me3 sites in *emf1*, *emf2*, and *fie* mutant plants. Because FIE is required during seed development and *fie* mutants are embryo-lethal, we used a transgenic line that expresses *FIE* only during the seed development stage to recover homozygous *fie* seedlings [18]. Relative to two-week old WT, plants impaired in each of these three genes have no petioles and rosette leaves, a short hypocotyl, and oval shaped cotyledons. *emf2* and plants impaired in *FIE* are similar in phenotype. The *emf1* allele used in this study, *emf1-2*, is a strong allele with a more severe phenotype than *emf2* ([32]; Figure 2C). Among the 7,751 genes marked by H3K27me3 in WT, 44% show reduced H3K27me3 in *emf1* mutants, 54% in *emf2*, and 84% in *fie* (Figure 2A, 2B, and 2D). This 84% H3K27me3 reduction is consistent with an earlier study [30], in which a 75% loss in a different *FIE*-impaired transgenic plant was reported. The loss of H3K27me3 in *fie* mutant seedlings indicates that H3K27me3 requires a functional PRC2 complex. The moderate decline of H3K27me3 in *emf2* could be due to partial replacement of EMF2 function by its homolog, VRN2 [15], [16]. The partial requirement for EMF1 shows that H3K27me3 is less dependent on EMF1 than on PRC2, indicating a site-specific EMF1-dependent H3K27 trimethylation. Nevertheless, 75% of the genes with reduced H3K27me3 in *emf1* have reduced H3K27me3 in *emf2* and *fie* (Figure 2E), indicating that trimethylation on these genes requires coordinated action by EMF1 and PRC2.

### Genes highly trimethylated on H3K27 and enriched for EMF1 binding

**Figure 3:**
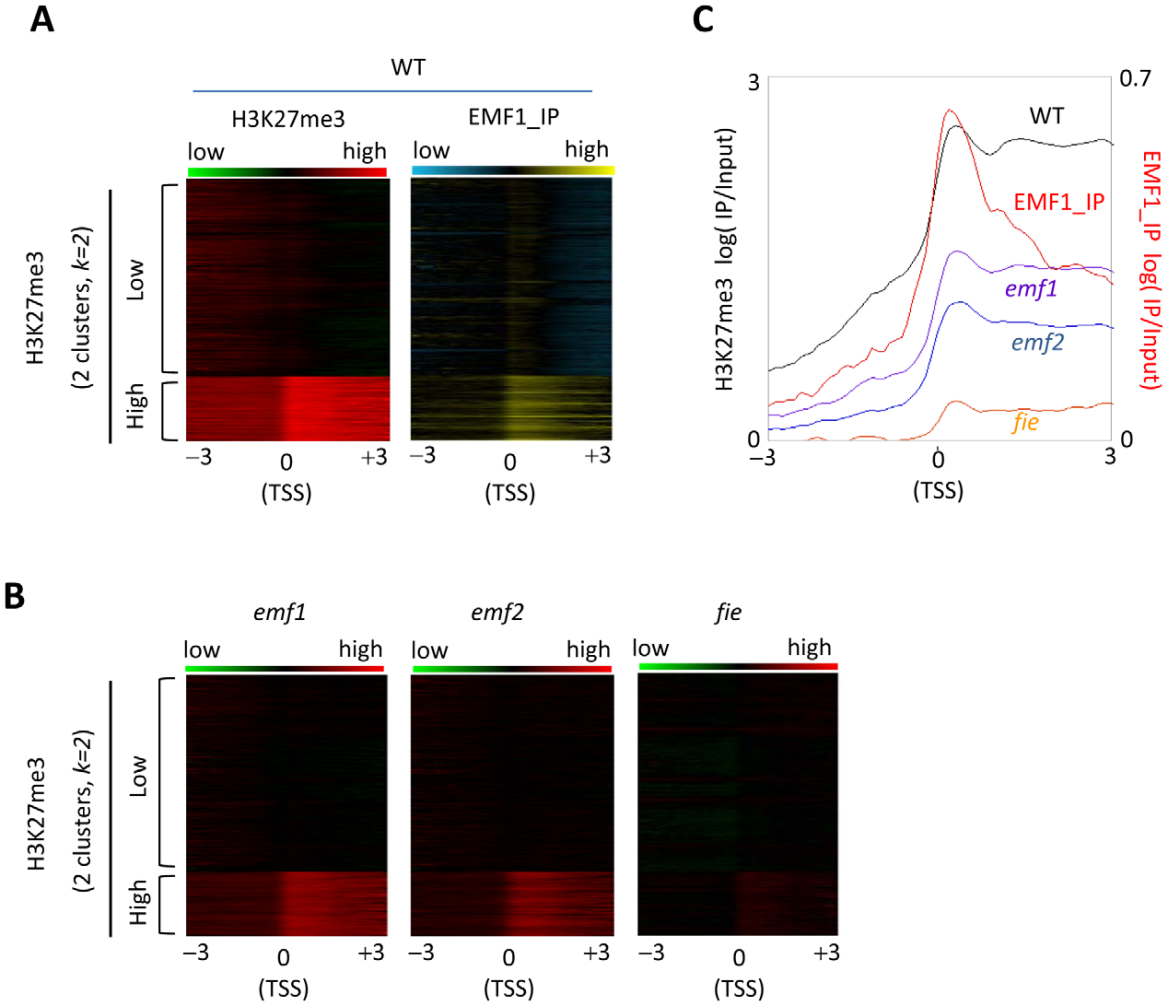
H3K27me3 modification and EMF1 binding pattern in genes of high and low H3K27 trimethylation levels. (A) Heat map of H3K27me3 and EMF1 binding patterns of the entire WT genome. All annotated sequences from the TAIR8 were aligned at the TSS and average signals for each 100 bp bin were plotted from 3 kb upstream of the TSS to 3 kb into the gene. Average H3K27me3 signal in each 100 bp bin in WT was calculated, and data sorted into two groups by an unsupervised *k-means* clustering algorithm (*k = 2*) and displayed as a heat map. The cluster of genes with highly trimethylated gene body is indicated with ‘High’ and the cluster of genes with low trimethylation in the gene body is indicated with ‘Low’. The left panel displays the H3K27me3 pattern, while the right panel displays EMF1 binding (EMF1_IP) dataset aligned according to the two clusters of H3K27me3 genes. (B) Heat map of H3K27me3 in three mutants. The H3K27me3 dataset of *emf1*, *emf2*, and *fie* were aligned according to the sorting order of H3K27me3 of WT. (C) The pattern of H3K27me3 in WT, *emf1*, *emf2* and *fie*, and EMF1 binding in WT in the cluster of highly trimethylated genes. The sequences of the genes with high H3K27me3 were aligned at TSS and average signals for each 100 bp bin were plotted from 3 kb upstream of the TSS to 3 kb into the gene.

Because peak calling necessarily involves arbitrary cutoffs, we supplemented the ChIPOTLe analysis that generated the H3K27me3 peaks by an unsupervised *k-means* clustering algorithm (*k* = 2, Figure 3A, left panel). The 28,244 Arabidopsis genes were aligned at the annotated TSS, the average H3K27me3 signal calculated in each 100 bp bin across the 6 kb region surrounding the TSS, and the data sorted into two clusters, high and low H3K27 trimethylation. High enrichment level of H3K27me3 in the transcribed, relative to the 5′ untranscribed, region is clearly seen in the highly trimethylated gene cluster (Figure 3A, left panel). We then arranged EMF1 binding strength to match the H3K27me3 sorting order (Figure 3A, right panel), and found that genes in the cluster of high H3K27me3 exhibit high enrichment level of EMF1 binding, while the cluster with low H3K27me3 genes show low enrichment level of EMF1 binding. We then arranged H3K27me3 levels in the three mutants according to the high and low H3K27me3 clusters (Figure 3B). The H3K27me3 level is most drastically reduced in *fie*, less in *emf2* and *emf1*, consistent with the ChIPOTLe analysis shown in Figure 2B.

Since the high H3K27me3 cluster of genes shows the most distinct pattern (Figure 3A and 3B), we plotting the average H3K27me3 signal and the EMF1 binding pattern of this cluster of genes across the 6 kb region surrounding the TSS in WT and in the 3 mutants (Figure 3C). The promoter regions of this highly trimethylated cluster of genes show minimal H3K27me3 modification, while it is highly enriched in the transcribed region. H3K27me3 enrichment is highest around the TSS, then declines slightly but is maintained throughout the 3 kb of the transcribed region. As expected, H3K27me3 enrichment is reduced in all three mutants, nearly absent in *fie* and partially lost in *emf2* and *emf1*. Despite the reduction in the mutants, the H3K27me3 pattern across the gene remains remarkably similar to WT. The EMF1 binding pattern of these highly methylated genes is similar to their H3K27me3 modification pattern in that EMF1 binds primarily the chromatin of the transcribed, rather than the promoter, region. However, EMF1 binding in this cluster of highly methylated genes shows a precipitous drop from the peak of binding at the TSS in the 3′ direction: the major binding is within 1 kb of the TSS (Figure 3C).

Results from the *k-means* clustering algorithm and the ChIPOTle method are consistent. We then used a Perl implementation of the ChIPOTle method to identify the EMF1-bound genes that are trimethylated and found 58% (3230) of the 5,533 EMF1-bound genes exhibit H3K27me3 peaks, called EMF1_K27 genes (p = 6×10^−184^; Fisher's exact test, see gene list in Table S2). Our subsequent analysis focused on the EMF1_K27 genes, highly trimethylated on H3K27 and enriched for EMF1 binding.

### Developmental functions of the EMF1_K27 genes

Gene ontology (GO) analysis of the 3230 EMF1_K27 genes revealed that EMF1 and H3K27me3 co-localize at a remarkably high number of genes involved in transcription factor activity, developmental pathways, and microRNA (miRNA) gene silencing (Table 1; Table S3). Relative to the whole genome, there is a 2.5–5 fold enrichment in the genes belonging to the categories of transcription factor activity, miRNA regulation, and genes involved in leaf, vascular, root, meristem, and flower development. EMF1 binds preferentially (p<0.05) genes involved in biotic and abiotic stresses and in the biosynthesis of, and response to, the major plant hormones: abscisic acid (ABA), auxin, brassinosteroids (BR), cytokinins (CK), ethylene, gibberellic acid (GA), jasmonic acid (JA) and salicylic acid (SA), and genes involved in biotic and abiotic stresses.

**Table 1:** Over-represented functional categories of EMF1-bound and H3K27 trimethylated genes.

	Genome	EMF1_K27	p-value		Genome	EMF1_K27	p-value
	(%)	(%)			(%)	(%)	
Transcription factor activity	3.1	17.4	3×10^−296^	ABA response & biosynthesis	0.4	0.8	2×10^−2^
Gene silencing by miRNA	0.1	0.4	6×10^−5^	Auxin response & biosynthesis	0.6	1.5	3×10^−8^
Leaf development	0.5	1.5	9×10^−13^	Ethylene response & biosynthesis	0.2	0.5	6×10^−3^
Vascular development	0.1	0.4	1×10^−6^	*GA response & biosynthesis	0.2	0.6	3×10^−5^
Root development	0.2	0.5	5×10^−5^	*JA response & biosynthesis	0.3	0.7	5×10^−6^
Cell wall development & biosynthesis	1.5	3.2	3×10^−11^	*SA response & biosynthesis	0.2	0.4	4×10^−2^
Cell morphogenesis & differentiation	0.3	1.2	8×10^−17^	*BR response and biosynthesis	0	0.2	2×10^−4^
Meristem development	0.1	0.4	1×10^−9^	Cytokinin response ans biosynthesis	0	0.2	2×10^−4^
Flower development	0.5	2.2	2×10^−26^	Response to abiotic stress	0.8	1.6	6×10^−5^
				Response to biotic stress	1.6	3.3	8×10^−11^

Over-represented GO categories in the 3230 genes bound by EMF1 and trimethylated on H3K27 (EMF1_K27) are grouped by function in development (detailed in Table S3) and shown.

*: GA: gibberellic acid, JA: jasmonic acid, SA: salicylic acid, BR: brassinosteroid.

**Figure 4:**
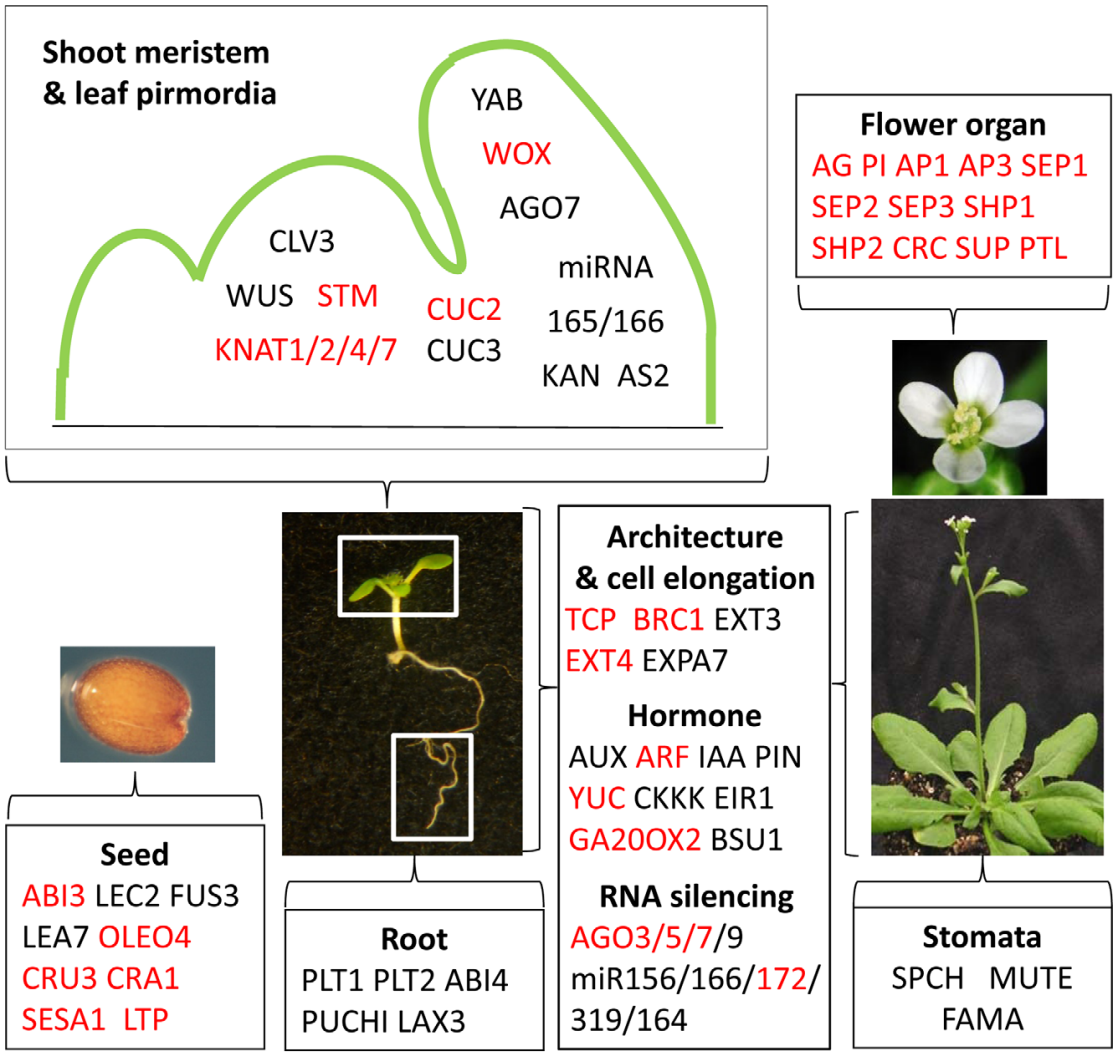
EMF1_K27 genes involved in Arabidopsis development. EMF1 represses most genes involved in seed and flower organ development in the seedlings. EMF1 is involved in localized expression of genes specifying shoot meristem, leaf polarity, root development, shoot architecture through direct interaction with the transcription factor and RNA silencing genes, and/or genes involved in hormone synthesis and action during vegetative development. Genes up-regulated in *emf1* mutants are marked red. *AS: ASYMMETRIC LEAF*; *AUX: AUXIN RESISTANT*; *ARF: AUXIN RESPONSE FACTOR*; *BSU1: BR SUPPRESSOR1*; *CLE:CLV3/ESR-RELATED*; *EIR1: ETHYLENE INSENSITIVE ROOT1*; *EXP: EXANSIN*; *IAA:INDOLE-3-ACETIC ACID INDUCIBLE*; *KAN: KANADI*; *KNAT: KNOTTED-LIKE FROM ARABIODOPSIS THALIANA*; *LAX3: LIKE AUX3*; *PIN: PIFORMED; PLT: PLETHERA; SESA1: SEED STORAGE ALBUMIN1; SPCH:SPEECHLESS; WOX: WUSHEL RELATED HOMEOBOX; YUC: YUCCA*. Full names of other genes are described in the text.

We next examined the annotated genes with known developmental functions (Figure 4; Table S4), beginning with the genes required for flower and seed development that are up-regulated in *emf1* mutants [33], [34]. We found that EMF1 binds a subset of these H3K27me3 modified genes (Table S4). For example, EMF1 binds 3 of the 4 major seed regulated genes marked by H3K27me3, namely, *FUSCA3* (*FUS3*), *ABA INSENSITIVE3* (*ABI3*) and 2 *LEAFY COTYLEDON2* (*LEC2*) [44], as well as, a fraction of the downstream seed maturation genes that are trimethylated, e.g., the *LATE EMBRYO ABUNDANT* (*LEA*), *OLEOSIN* (*OLEO*), and *LIPID TRANSFER PROTEIN* (*LTP*), and seed storage protein genes (Table S4). It is worth noting that some genes in the same families are bound by EMF1 but are not marked with H3K27me3 (Table S4).

EMF1 silences the flower developmental program by interacting with and repressing all known flower organ identity genes and other genes specifying flower organ development, e.g., *CRC*, *SUPERMAN* (*SUP*), and *PETAL LOSS* (*PTL*, [45], [46]; Figure 4). Flower organ identity genes are all type II *MADS box* genes [47]. We found that EMF1 preferentially interacts with type II *MADS box* genes. EMF1 does not interact with the Type I *MADS box* genes that are important for female gametophyte and early seed development, e.g., *PHE1* (*AGL37*), *PHE2* (*AGL38*), *AGL23*, and *AGL61* [48], [49], although they are H3K27 trimethylated in Arabidopsis seedlings (Table 2; Table S4).

**Table 2:** *MADS box* genes marked by H3K27m3 only and EMF1_K27 genes.

EMF1_K27				aH3K27me3 only
Group I			Group II	
Up-regulated in *emf1*	No expression change in *emf1*		No expression change in *emf1*	No expression change in *emf1*
Type II	Type I	Type II	Type I	Type I
*MADS box* genes	*MADS box* genes	*MADS box* genes	*MADS box* genes	*MADS box* genes
*AG*	*AGL50*	*AGL17*	*AGL41*	*PHE1/AGL37*
*AGL15*	*AGL86*	*AGL19*	*AGL49*	*PHE2/AGL38*
*AGL71*	*AGL92*	*AGL20*	*AGL82*	*AGL23*
*AP3*	*AGL96*	*AGL24*	*AGL83*	*AGL61*
*AP1*	*AGL57*	*AGL44*		*AGL96*
*STK*	*AGL97*	*AGL6*		
*PI*		*AGL67*		
*SEP2*		*AGL12*		
*SEP1*		*AGL13*		
*SEP3*		*AGL8*		
*SHP2*		*AGL1*		
*AGL42*		*CAL*		
*AGL14*				
*TT16*				
*FLC*				

a H3K27me3 only—H3K27me3 marked genes that are not EMF1-bound.

Vegetative development requires not only the repression of the seed and flower programs but also dynamic activation and repression of genes to specify cell fates in the meristems and to dictate organized cell growth and differentiation. Our study of seedling chromatin showed that EMF1 binds H3K27me3 marked genes that specify cell fates in shoot and root apices and control leaf polarity, e.g., *SHOOT MERISTEMLESS* (*STM*), *CLAVATA3* (*CLV3*), and *WUSHEL* (*WUS*) (Figure 4). Shoot meristem and leaf primordia in the shoot apex are separated by the expression of the boundary-specific genes encoding the NAC domain transcription factors, *NO APICAL MERISTEM* (*NAM*) and *CUP SHAPED COTYLEDONE* (*CUC*) [50], [51], [52], which are negatively regulated by the *TEOSINTE BRANCHED1*, *CYCLOIDEA*, and *PCF* (*TCP*) genes. *NAM*, *CUC2*, and *CUC3* are all trimethylated and bound by EMF1 (Figure 4). EMF1 interacts with 9 of the 10 H3K27 trimethylated *TCP* genes. *TCP14* affects internode length and leaf shape [53]. EMF1 interaction with *TCP* genes that affect diverse aspects of Arabidopsis shoot growth and architecture is consistent with the pleiotropic effect of *EMF1* impairment on Arabidopsis shoot development that includes petiole-less cotyledons, short hypocotyl and short inflorescence stem, due to limited cell elongation in *emf1* mutants [31].

Hormones mediate growth and differentiation after germination. H3K27me3 marks a full spectrum of genes involved in indole-3-acetic acid synthesis, transport and signaling [54], most of them are EMF1-bound (Figure 4; Table S4). EMF1 also interacts with many other hormone genes marked with H3K27me3, e.g., *CYTOKININ OXIDASE* (*CKKX*), *GA OXIDASE*, and genes involved in JA, BR, and ethylene synthesis and response (Table S4). Temporal and spatial regulation of these EMF1_K27 genes is critical for normal shoot and root architecture and growth patterns.

MicroRNA (miRNA) regulation of target genes controls various aspects of developmental transitions [55]. The juvenile to adult transition of the vegetative shoot is coordinated by the antagonistic activities of miR156 and miR172, through their opposite expression pattern and the antagonistic function of their target genes [56]. The miR319-TCP and miR164-CUC miRNA-target nodes are involved in regulated cell proliferation during leaf morphogenesis [55]. EMF1 interacts with about 50% of the miRNA genes marked by H3K27me3 (Table S4). The *AGONOUTE* (*AGO*) genes mediate gene silencing through small RNA-directed RNA cleavage and translational repression [57]. EMF1 interacts with all H3K27 trimethylated *AGO* genes, including *AGO10/ZIWILLE* (*ZLL*) (Table S4; Figure 4), which acts in the siRNA and miRNA pathways and is essential for multiple developmental processes in plants [57]. Thus EMF1 may mediate juvenile and adult growth, as well as, lateral organ enlargement through the regulation of *AGO* and miRNA genes.

In summary, to promote vegetative development and to regulate cell differentiation during shoot and root organogenesis, EMF1 binds genes required for other developmental phases and genes specifying cell identities. These are primarily genes trimethylated by EMF2-PRC2 on their H3K27.

### EMF1-dependent and -independent EMF1_K27 genes differ in function and average transcription score

**Figure 5:**
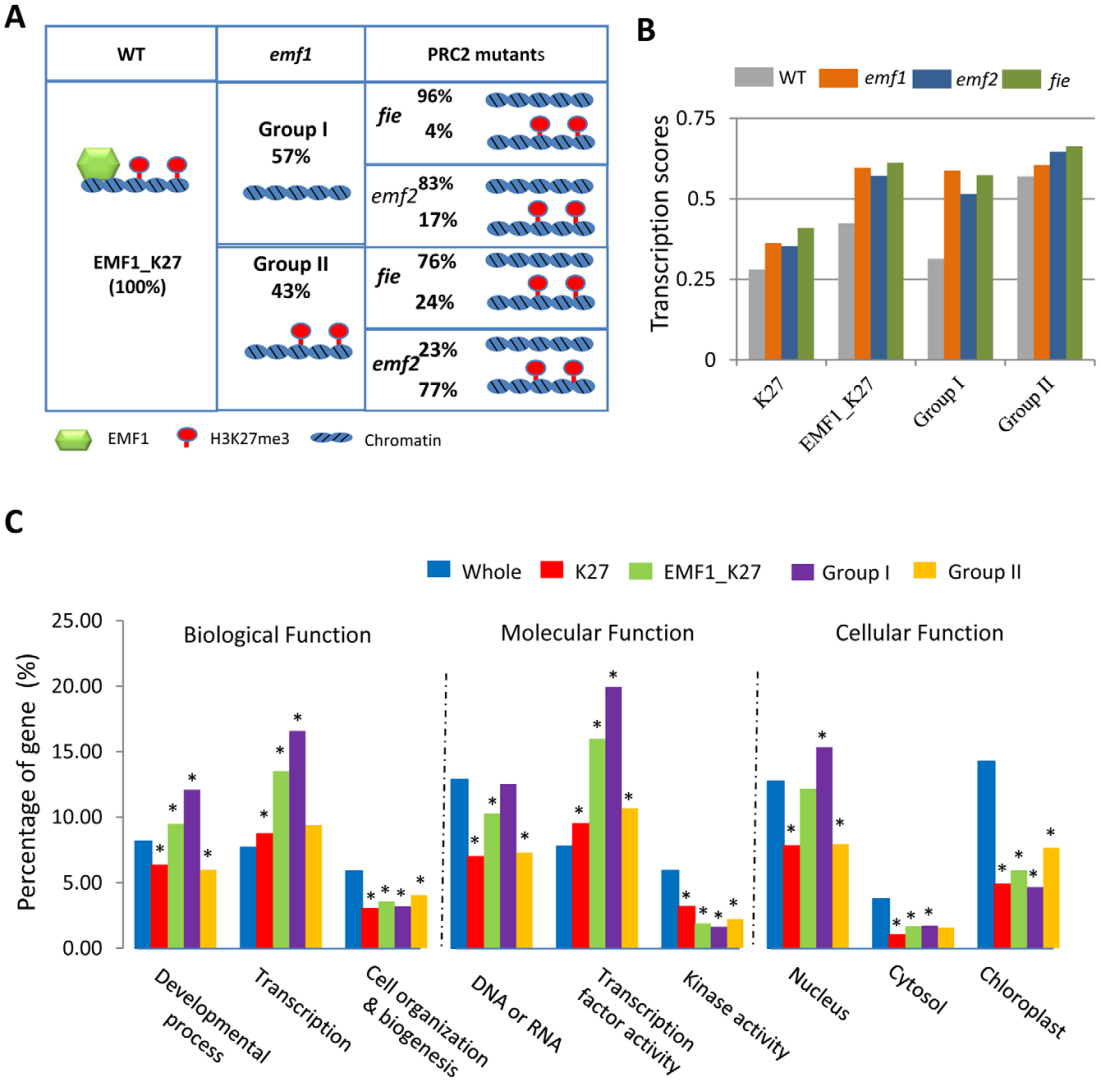
Functional analysis of groups of genes based on EMF1 binding and H3K27me3. (A) H3K27me3 status of EMF1-bound genes in *emf1* and PRC2 mutants. In WT, 3,230 (100%) were EMF1-bound and trimethylated on H3K27 (EMF1_K27). Among them, 1,845 or 57% of the EMF1_K27 genes, Group I, showed reduced H3K27me3 in *emf1* mutant. Among the 1,845, 1,536 (83%) and 1,783 (96%) showed reduced H3K27me3 in *emf2* and *fie* mutants, respectively. 17% and 4%, however, were trimethylated in *fie* and *emf2*, respectively. 1,385 or 43% of EMF1_K27 genes, Group II, remained trimethylated in *emf1*. Among the 1,385 genes remained trimethylated in *emf1*, 24% and 83% also maintained, while 76% and 17% showed reduced, methylation in *fie* and *emf2*, respectively. (B) Average transcriptional score of genes of H3K27 trimethylated (K27), EMF1_K27, Group I, and II in WT and 3 mutants. Each feature or hybridization signal on the NimbleGen array is represented as log2-ratio of the genomic DNA for the amplified cDNA from the sample. After median normalization, each feature was annotated and scored using perl scripts to produce the transcription score. Y- axis: arbitrary units representing average transcription scores [log2 (mRNA signal intensity)] for genes in given groups. (C) Gene ontology (GO) annotation analysis. Percent representation of whole genome (whole), trimethylated (K27), EMF1_K27, Group I, and Group II based on the 9 GO categories, 3 in each of the biological, molecular and cellular functional category is detailed in Table S5. Y-axis: the percentages of genes in each GO category. Categories marked with “*” have a p-value<10^−3^.

We examined H3K27me3 of the EMF1_K27 genes in *emf1* mutants and found two groups of genes. Group I genes–the EMF1-dependent H3K27me3 genes – comprising 57% of the EMF1_K27 genes (1845/3230), are not H3K27me3 enriched in *emf1* mutants. Group II genes– EMF1-independent H3K27me3 genes– comprising 43% of EMF1_K27 genes' are trimethylated in *emf1* mutants (Figure 5A; Table S2).

To determine whether the H3K27me3 of EMF1-bound genes is mediated by PRC2, we examined trimethylation in *fie* and *emf2* mutants. Most EMF1-bound genes showed reduced methylation in *fie* –96% of Group I and 76% of Group II genes (Figure 5A). Therefore, both Group I and Group II genes are indeed methylated by PRC2. 83% of Group I and 23% of the Group II genes showed reduced methylation in *emf2*. Methylation may be less affected in *emf2* than in *fie* because of *EMF2* and *VRN2* redundancy, while FIE participates in both EMF2- and VRN2-PRC2.

#### Group I and Group II genes differ in their average RNA abundance/transcription score in WT, and in their dependency on EMF1 and PRC2 for repression

(Figure 5B) Transcriptome analysis of WT and the 3 mutant seedlings was carried out using the same NimbleGen HD2 tiling arrays and samples employed for H3K27me3 analysis. The average transcription score, defined as the average transcript level of all the genes in a given category, of the WT and mutant samples were obtained for the 7,751 highly trimethylated (K27), the EMF1_K27, the Group I and the Group II genes (Figure 5B). In WT seedlings, Group I genes have a lower average transcription score than Group II genes. Moreover, the average transcription score of Group I genes is increased in *emf1* mutants by 47%, in *emf2* by 39% and in *fie* by 45%. Up-regulation of Group I genes in all three mutants demonstrates their repression by EMF1 binding and H3K27 trimethylation. This phenomenon is observed, to a lesser extent, in the larger group of 7,751 K27 and 3,230 EMF1_K27 genes. Group II genes, which, on average, encode genes of higher mRNA abundance than Group I, showed limited up-regulation in the 3 mutants.

#### The Group I and Group II genes differ somewhat in their functions

Genes involved in developmental and transcription processes and encoding transcription factors and proteins located in the nucleus are highly overrepresented in Group I, while genes involved in cell organization/biogenesis, kinase activity, and cytoplasmic and chloroplast functions are underrepresented (Figure 5C). In Arabidopsis, genes involved in developmental processes comprise about 8.18% (2,310/28,244) of the genome. During seedling growth, genes needed for seedling development are active. Genes responsible for other stages of development are probably silenced by trimethylation at their H3K27. 6.33% of K27 genes, 9.47% of EMF1_K27 genes, and 12.1%, of the Group I genes are involved in developmental processes (Figure 5C; Table S5). The same trend, increasing representation from the K27 to EMF1_K27 to Group I genes, is seen with genes involved in transcription processes and encoding proteins located in the nucleus, but not genes involved in cell organization processes and encoding proteins located in cytosol or the chloroplast. Only 6% of Group II genes are involved in developmental processes. Using the EMF1_K27 *MADS box* gene targets as an example, Group I genes contain many more *MADS box* genes, particularly the Type II *MADS box* genes; while Group II has fewer *MADS box* genes and all of them are Type I (Table 2; Table S4).

#### Genes of known developmental functions are repressed by EMF1 and PRC2

Analysis of the NimbleGen transcriptome data performed in this study revealed that many of the genes with important developmental function mentioned in the previous section are up-regulated in *emf1* mutants. Examples for the Group I genes are the flower organ identity genes and the seed regulatory and maturation genes (Figure 4). Cell fate determination genes, such as the *KNAT1*, *KNAT4*, *KNAT7*, *HOMEO BOX21* (*HB-21*), *STM*, *WOX2*, and *BEL1- LIKE HOMEODOMAIN8* (*BLH8*), *miRNA* (*166A, 166E, 172D*), *AGO5*, *AGO9*, and the boundary-specifying genes, *TCP1*, *TCP17*, *BRC1*, *CUC2*, and *NAM*, are also up-regulated in *emf1* (Figure 4; Table S6). We detected up-regulation of the hormone synthesis gene *GA20OX3* in *emf1* mutants. Examining previously published GeneChip data [34], we found additional Group I genes up-regulated (Table S6), e.g., *FLC*, *KAN1*, *KAN2*, *SHP1*, *ULT1*, *YUC4*, *WOX8*, *WOX11*, *WOX12*, and *BLADE ON PETIOLE1* (*BOP1*). BOP1 is required for repressing meristem activity at the cotyledon base [58] and promoting floral meristem identity [59].

Many Group II genes also depend on EMF1 for repression. Examples include the 6 seed maturation genes and *JASMONATE-ZIM-DOMAIN PROTEIN 1* (*JAZ1*, the repressor of JA signaling, [60]) (Figure 4; Table S6). Examination of previously published GeneChip data revealed additional Group II genes up-regulated in *emf1*, including *ARF21*, *ARF23*, *CLE21*, *EXT4*, and *AtGA20OX2*. *CLE21* misexpression would result in a miniature shoot [61], [62]. EMF1 repression of *CLE21* is consistent with normal shoot growth.

We then examined whether the above EMF1 repressed genes are regulated by PRC2. Overall, 80% of the Group I and II genes up-regulated in *emf1* are also up-regulated in *fie* or *emf2* mutants (Table S6), indicating that most of these genes are indeed regulated via the PcG mechanism.

A large number of Group I and II genes did not change expression in the mutants. This is probably due to localized expression and/or low transcript level. With microarrays, differential expression in such genes is difficult to detect in RNA extracted from whole seedlings. A slight up-regulation of *KNAT6*, a meristem-specific gene, in plants impaired in PRC2 genes [54] and of *VERNALIZATION INSENSITIVE3* (*VIN3*) in plants impaired in *EMF1* [63] can be detected by RT-PCR, but not microarrays. Additionally, EMF1 may not be solely responsible for repression or activators may be needed for expression.

### EMF1 regulation of PcG and trxG genes and autoregulation

EMF1 targets many chromatin protein genes marked by H3K27me3 in WT seedlings, including *FIS2*, *VRN2*, *MEA*, *ULTRAPETALA1* (*ULT1*), and *ULT2* [64]. ULT1 is a component of trxG, the complex that antagonizes PcG action. EMF1 binding apparently represses *ULT1*, as its transcription is up-regulated in *emf1* mutants (Table S6). EMF1 does not bind the chromatin of *EMF2* or the PRC1-like components, *LHP1*, *AtBMI1A*, *AtBMI1B*, *AtRING1A*, and *AtRING1B* (Table S2; [41], [65]), which are required during postembryonic development, as is EMF1. EMF1 does bind *AtRING1C*, an imprinted gene expressed in the endosperm [66].

**Figure 6:**
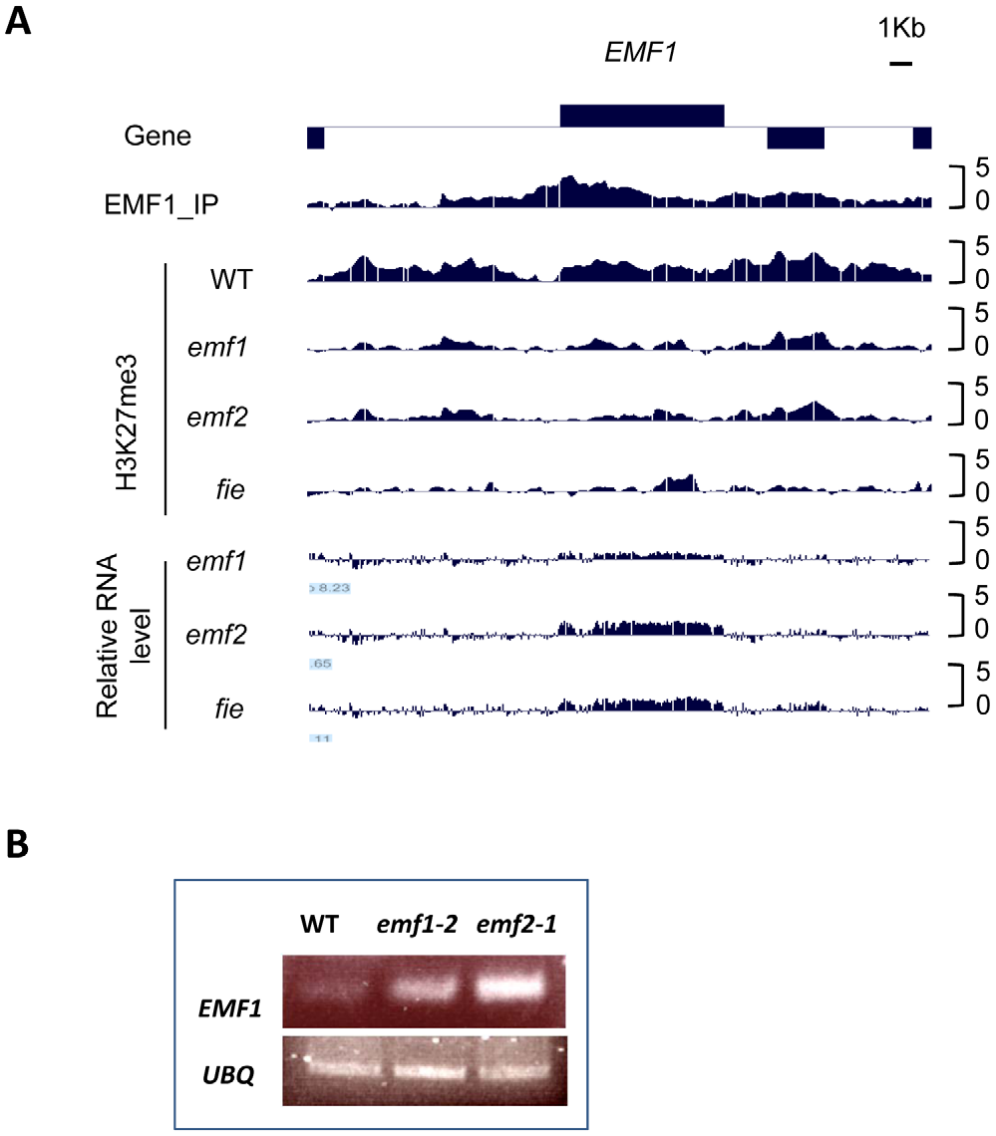
PcG-mediated EMF1 autoregulation. (A) EMF1 binding and H3K27me3 marked sites on *EMF1* chromatin in WT seedling, followed by H3K27me3 modification and expression change of *EMF1* in *emf1*, *emf2* and *fie*. (B) Reverse transcription-PCR (RT-PCR) analysis of *EMF1* expression in *emf1*, *emf2*, and WT fourteen days after germination, using *UBIQUITIN* (*UBQ*) as the loading control.

To investigate the epigenetic regulation of *EMF1*, we examined EMF1 interaction with itself. Interestingly, EMF1 binds its own chromatin strongly. Figure 6 shows EMF1 enrichment of the transcribed region of the *EMF1* gene (p<10^−20^). This high level of EMF1 enrichment on *EMF1* chromatin is accompanied by H3K27 trimethylation in WT, which is reduced in *emf1* mutants, thus placing *EMF1* in the category of Group I genes. Furthermore, *EMF1* is up-regulated in *emf1* mutants (Figure 6), providing evidence of EMF1 autoregulation. EMF1 transcript level is also elevated in *emf2* and *fie* mutants, indicating its repression via a PcG-mediated mechanism.

### EMF1 regulates highly transcribed genes without H3K27me3

In addition to the EMF1_K27 genes, we investigated the 2303 EMF1-bound but not trimethylated (EMF1_no_K27) genes in WT seedlings to find out their functional categories and whether they are regulated by EMF1 and PRC2 (Table S2). GO analysis showed that the fraction of genes involved in transcription and developmental processes and genes encoding transcription factors is lower in the EMF1_no_K27 than the EMF1_K27 genes, while genes involved in cellular organization and biogenesis, cytosol, and chloroplast are over-represented in the EMF1_no_K27 genes (Table S5). The EMF1_no_K27 genes tend to be actively transcribed genes with high RNA levels. Their average transcript score is more than 4 times that of the EMF1_K27 genes –1.83 for the EMF1_no_K27, relative to 0.42 for the EMF1_K27, genes.

Analysis of NimbleGen transcriptome data showed about 14% of the EMF1_no_K27 genes is up-regulated and 7% down-regulated in *emf1* mutants. A high percentage of these genes are similarly up- and down-regulated in the *emf2* and *fie* mutants, indicating a coordinated regulation of these genes by EMF1 and PRC2 (Figure S4A).

We have previously shown that many photosynthesis genes that encode chlorophyll a/b binding proteins and photosystem I and II proteins are down-regulated in *emf1* and *emf2* mutants [33], [34]. Seventy two percent of these genes are EMF1-bound [34], which are all EMF1_no_K27 genes and many are coordinately down-regulated in all three mutants (Table S2; Figure S4A and S4B). These results suggest that EMF1 activates their expression in the absence of H3K27me3. Indeed, PRC1 in fly and vertebrate are in some cases recruited to the target genes independent of PRC2 or H3K27me3 [67], [68]. Alternatively, despite EMF1-binding, deregulation of these genes in *emf1*, *emf2*, and *fie* mutants are a consequence of severe phenotypic aberrations in response to loss of these central regulators of development.

## Discussion

We set out to understand the function of *EMF1*, a plant specific gene, and its role in PcG-mediated gene regulation. To this end we generated genome-wide maps of EMF1 binding, and examined H3K27me3 modification and transcription on the EMF1-bound genes in WT, *emf1*, and plants impaired in PRC2 components. Analysis of a large number of target genes allowed us to ascertain a wide range of EMF1 functions. The data suggest that EMF1 regulates gene activity via diverse mechanisms. How the EMF1-bound, but unmethylated, genes are regulated is unclear at this moment. But the EMF1_K27 genes are most likely regulated via the classical PcG mechanism. Recent findings indicate that PRCs have multiple potential modes of action that go beyond the classical hierarchical model of synergistic effects of PRC2 and PRC1 [13]. In mammalian cells, opposing effects of PRC1 and PRC2 on gene activity have been observed and PRC1 can be recruited independently of PRC2-mediated gene silencing [69]. To further characterize the role of EMF1 in the PcG-mediated epigenetic mechanism, we focused our investigation on the EMF1_K27 genes, studying the requirement of EMF1 for H3K27me3 and the impact of EMF1 and PRC2 on gene expression.

### EMF1 functions as a Polycomb Group protein

Our genome-wide study provided new lines of evidence that support EMF1 acting via the PcG mechanism. First, EMF1 interacts mostly with euchromatic sites located on all 5 chromosomes, a pattern similar to H3K27 trimethylation. Second, on the genic level, the EMF1 binding pattern mimics that of the H3K27me3 in binding the transcribed, not the promoter, region with the peak binding activity at the 5′ TSS. Third, EMF1 represses the seed and flower development genes and cell fate determination genes that are also modified by H3K27me3. Fourth, H3K27 trimethylation on EMF1-bound genes is mostly dependent on PRC2 and gene expression is coordinately regulated by EMF1 and PRC2. These findings demonstrate that, for genes that are highly enriched for EMF1 binding and H3K27me3, EMF1 functions in the PcG mechanism.

### Role of EMF1 in the PcG mechanism

We investigated H3K27me3 dependency on EMF1 binding and found two groups of genes. Group I genes are richer in transcription factors and their repression is more dependent on EMF1 and PRC2 than Group II genes. Most importantly, Group I genes are dependent on EMF1 for H3K27me3 modification, while Group II genes are not. For Group I genes, which require EMF1 for K27me3, EMF1 may act prior to, or as a member of, PRC2 to trimethylate H3K27. For Group II genes that do not require EMF1 for H3K27me3, EMF1 may have a PRC1 function, or may be unrelated to PcG action. Since many Group II genes require PRC2 for H3K27me3, EMF1 is likely to act via the PcG mechanism, functioning downstream of H3K27trimethylation, as does PRC1.

The characteristics of the four Arabidopsis RING-finger proteins, AtRING1A, AtRING1B, AtBMI1A, and AtBMI1B, are consistent with their functioning like the mammalian PRC1 uibquitin ligase, which monoubiquitinates H2AK119 [8], [37]. EMF1 interacts with these proteins, and is required for these RING-finger proteins' monoubiquitination of H2AK119 (H2Aub), thus implicating EMF1 in PRC1 activity. The RING-finger proteins also interact with CLF [40], the PRC2 H3K27 trimethylase, and with LHP1 [37], [41], [70]. The EMF1 binding pattern is similar to that of H2Aub in mouse embryonic fibroblast cells [71] in that both EMF1 binding and H2Aub localization are enriched in the 1 kb 5′ coding region. It is proposed that H2A ubiquitination interferes with early transcript elongation [67]. EMF1 preferential localization in the 5′ coding region is consistent with its involvement in PRC1's role in blocking transcription elongation by preventing RNA polymerase movement through the compacted nucleosomes [67].

However, EMF1 appears to partner with these RING-finger proteins only on a select group of target genes. Most notably, the signature EMF1 targets, the flower organ identity genes *AG* and *AP3*, are not regulated by the 4 Arabidopsis RING-finger proteins. However, the class I *KNOX* (*KNOX1*) genes, including *STM*, *KNAT1*, *KNAT2*, and *KNAT6*, as well as *WUS*, and the seed regulator, *FUS3*, are negatively regulated by both EMF1 and the RING-finger proteins [37], [40]. EMF1 is bound to all these genes in Arabidopsis seedlings. Their ectopic expression in loss-of-function mutants suggests that these genes are direct target genes of the RING-finger proteins. Interestingly, their H3K27me3 shows varying degrees of dependence on EMF1. *KNOX1* and *WUS* are Group I genes: H3K27 trimethylation depends on EMF1. *FUS3* belongs to Group II: EMF1-independent H3K27me3 (Table S2).

EMF1 may act on Group II genes such as *FUS3* by assisting the PRC1 activity of the RING-finger protein-LHP1 complex following H3K27 trimethylation by PRC2. For Group I genes such as *STM*, EMF1 may participate in each PcG complex separately or may act like a linker protein that assists PRC2 in spreading H3K27me3, while helping PRC1 monoubiquitinate H2A. EMF1 interaction with MSI1 [36] and with the RING-finger proteins [37] is consistent with its involvement in both PRC2 and PRC1 activities. CLF interacts with AtRING1A/1B in yeast 2-hybrid assays [40]. Our results, together with this finding indicate a close association of PRC2 and PRC1 in Arabidopsis. This might be indicative of evolutionary divergence of PcG mechanisms. In Drosophila PRC2 and PRC1 are separate functions. Our study indicates that in Arabidopsis PcG proteins can also participate in closely linked PRC2-PRC1 function.

### EMF1 enhances the PRC2 targeting of developmental genes

EMF1 and the PRC2 proteins have a different evolutionary history [72], [73]. The PRC2 ancestral sequences existed prior to the divergence of the animals and plants. During plant evolution, gene duplication generated alternate PRC2 components that diversified to control different functions. *EMF1* is a plant-specific gene with homologous sequences found only in higher plants. It might have functioned first as a general transcriptional regulator for genes involved in development and basic cellular and biochemical activities. Coupling EMF1 with H3K27 trimethylation could have led to an enhanced targeting of genes in development. The repression of flower development, which effectively lengthens the vegetative phase, coupled with elaborating plant architecture through the regulation of hormone and signaling genes, may have been instrumental in the evolution of organisms with multiple developmental phases and diverse signaling processes. This is suggested by the progressive increase in the representation of genes involved in transcription and developmental processes from the H3K27me3 modified genes to the EMF1_K27 to Group I genes (Figure 5C; Table S5). The fact that EMF1_K27 genes are highly enriched with H3K27me3 and EMF1 binding suggests an emphasis on this epigenetic mechanism through robust retention of repressive chromatin during cell differentiation. Similarly, in mammalian cells, some genes are controlled by PRC1, independent of PRC2, and others are coordinately controlled by PRC1 with PRC2 [13]. The vast majority of developmental regulator genes are bound by both PRC1 and PRC2, while genes bound by only one PRC are enriched for the membrane proteins [12].

### EMF1 regulation of PcG and trxG genes, and autoregulation

The similarity of mutant phenotypes suggests that EMF1 acts primarily with EMF2-PRC2 to mediate developmental processes in Arabidopsis. EMF1 also acts together with AtBMI1A/1B and AtRING1A/1B to regulate genes maintaining cell identity. This means that EMF1 should not silence the EMF2-PRC2 or the 4 RING-finger protein genes. Indeed, EMF1 does not target *CLF*, *EMF2*, *SWN*, or the RING-finger protein genes. EMF1 does not interact with *VRN2* either, which has similar, ubiquitous expression patterns as *EMF1* and *EMF2*. EMF1 interacts with the chromatin of *FIS2* and *MEA*, components of FIS2-PRC2, consistent with their inactivity after germination. So far, no up-regulation of these two genes has been detected in the absence of EMF1. Thus, EMF1 binding may not be the sole factor responsible for their repression, or their expression may require activators that are absent after germination.

EMF1 coordinates only with EMF2-PRC2 to regulate PcG target genes. Neither EMF1 nor EMF2–PRC2 regulate the Type I *MADS box* genes involved in female gametophyte and endosperm development (Table 2), including *PHE1* and *PHE2*, whose maternal inheritance is mediated by FIS2-PRC2 [74]. *PHE1* and *PHE2* do not interact with EMF1 and are not normally expressed post-germination. Their repression is not likely dependent on EMF1 or EMF2–PRC2, for they are not ectopically expressed in *emf1* and *emf2* mutants, even though they are trimethylated on H3K27 (Figure S3). This is consistent with a close association of EMF1 with EMF2-PRC2 and its lack of involvement in FIS2-PRC2 mediated epigenetic repression.

ULT1 interacts with ARABIDOPSIS TRITHORAX 1 (ATX1), thus is considered a component of the Arabidopsis trxG that acts to antagonize PcG action, as evidenced by *ult1* mutants rescuing the *clf* mutant phenotype [75]. *ULT1* and *ULT2*, a homolog of *ULT1*, are EMF1_K27 genes (Table 2), and considered to be anti-repressors of *PcG* genes. *ULT1* is up-regulated in *emf1* and *emf2* (Table S6), and both *ULT1* and *ULT2* are up-regulated in *fie* [30]. The temporal and spatial differentiation of *ULT1* and *ULT2* expression patterns is likely to involve EMF1, but its role in the fine tuning of the repressor and anti-repressor balance in regulating gene expression remains to be characterized. Similarly, EMF1 autoregulation must be a dynamic process in order to modulate its epigenetic regulatory activities at a cellular level. Indeed, although *EMF1* transcripts and proteins have been found in all tissues and organs [28], [34], [36], their expression pattern differs temporally and spatially in WT and *emf1* plants [31]. This may result from EMF1's autoregulatory actions. Future investigation of cell- and tissue-specific EMF1 binding activities is needed to address these questions.

### EMF1_K27 genes maintain vegetative developmental phase and cell identity

Temporally, EMF1_K27's major role, as plants undergo seed, vegetative and reproductive phase transitions, is to maintain repression of the seed and flower genes so as to allow vegetative growth after germination. Thus, major seed regulators and flower organ identity genes are repressed. Spatially, EMF1 is involved in switching or maintaining differentiated cell states, such that EMF1 probably represses the leaf polarity genes, *KANADI* and *YABBY*, and the shoot meristem-specific genes, *STM* and *KNAT2*, in the differentiated leaf, hypocotyl and root cells. The three genes specifying stomata development, *SPCH*, *FAMA*, and *MUTE*, are inactivated in most cells except during stomata differentiation in the leaves. *PLT1* and *PLT2* are silenced in the shoot and mature root so that meristematic growth is restricted to the root tip. Future investigation of gene expression in separated tissues or in situ assays on individual genes of interest will clarify the role of EMF1 binding on the regulation of genes that did not show apparent expression change in mutants.

## Materials and Methods

### Plant materials and growth conditions

WT and *emf* mutants, *emf1-2* and *emf2-1*, of Arabidopsis used in this study are from the Columbia ecotype background, and have been described [33]. The transgenic plants impaired in *FIE* was described in Kinoshita et al., [18], and the *pEMF1*::3*FLAG*-tagged *EMF1*, called RM, in Calonje et al., [36]. Seeds were surface-sterilized and plated on agar plates containing 2/5X strength Murashige and Skoog medium [76]. The plates were placed for 2 days at 4°C and then transferred to a short day growth room (8 hrs light/16 hrs dark) at 21°C. WT, mutants, and transgenic plants were harvested after growth for 14 days for expression and ChIP experiments.

### ChIP and microarray assays

ChIP experiment was performed according to published procedure [36] on WT, *emf1*, *emf2*, transgenic *FIE*, and transgenic plant harboring the *EMF1-3FLAG* construct grown in the short day growth condition for 14 days. Due to homozygous lethality of *emf1*, *emf2*, transgenic *FIE* mutants, seeds from heterozygous plants were germinated; mutants were separated from the WT-looking plants and harvested. Plants were vacuum infiltrated in 1% formaldehyde solution for half an hour to cross-link the chromatin. Tissues were ground in liquid nitrogen, nuclei isolated, and chromatin extracted according to Bowler et al., [77]. Chromatin was sheared by sonication (Microson, MS-50), 10″ on and 10″ off for 10 times to generate 0.5- to 2 kb fragments. For immunoprecipitated chromatin (IP), monoclonal anti-FLAG mouse antibody (Sigma F1804) and polyclonal anti-H3K27me3 antibody (Upstate, rabbit IgG, 07-449) were added to fragmented chromatin to precipitate EMF1-bound and H3K27me3 modified chromatin, respectively. The cross-linking of IP was reversed with 5M NaCl and DNA precipitated by 100% EtOH. For the Input control (Input), 0.5% of total chromatin before immunoprecipitation was reverse cross-linked by 5M NaCl and DNA isolated by 100% EtOH. The relative amount of DNA was determined by PCR and spectrophotometry (NanoDrop, ND1000).

ChIP-chip was performed according to the NimbleGen protocol (Roche, www.nimblegen.com). IP and Input DNA were amplified using the Whole Genome Amplification kit (Sigma, GenomePlex Kit, WGA2), and labeled with CY5 and CY3, respectively [78], [79]. Combined samples, which include 10 ug of CY5-labeled IP and 10 ug of CY3-labeled Input DNA, were hybridized with NimbleGen HD2 arrays (http://www.nimblegen.com/products/chip/custom/index.html), with 2.16 million, ∼50mer probes that allow coverage of the entire Arabidopsis genome without gaps. The hybridization and data extraction were performed at the Fred Hutchinson Cancer Research Center DNA array facility (http://www.fhcrc.org/science/shared_resources/genomics/index.html). Microarray hybridization was repeated three times with independent biological samples.

For global gene expression studies, total RNA was extracted from 14 old WT and mutants using trizol (Invitrogen) and converted into cDNA according to Moon et al., [33]. Genomic DNA from WT and cDNA from mutants and WT were labeled with CY3, and CY5, respectively, and combined to hybridize with NimbelGen HD2 arrays as described.

### Data analysis

For microarray analysis, signal intensity data of microarrays are extracted from the scanned images of each array using NimbleScan, NimbleGen's data extraction software. For ChIP-chip data, each feature on the array is represented as log_2_-ratio of the input signals for the immunoprecipitated DNA. The log_2_-ratio is computed and scaled to center the ratio of data around zero. Peaks were derived using a Perl implementation of ChIPOTle (https://sourceforge.net/projects/chipotle-perl/) [80] using a window size of 300 bp, step size 50 bp with specific cut-offs. The p-value of 1×10^−35^ and the peak length of 300 bp were applied as a cut-off for dataset of H3K27me3, and the p-value of 1×10^−6^ and the peak length of 100 bp were applied as a cut-off for the EMF1 binding dataset, respectively. Peaks were annotated using TAIR 8. Genome browser views were generated using the SignalMap software from NimbleGen. End analysis was done as described in Zilberman et al., [79]. For RNA expression data, each feature on the array is represented as log_2_-ratio of the genomic DNA for the amplified cDNA from each mutant. After median normalization, each feature was annotated and scored using perl scripts. To find differentially expressed genes in mutants, the datasets of [mutant –WT] were generated by subtracting probe values in the mutant datasets from counterpart values in the WT datasets, and then, *arbitrary cutoff* of ±1.5sd was used to select differentially regulated genes.

### Gene Ontology analysis

The functional categories of target genes were assigned based on the GO annotations from the TAIR website [81]. For functional categories of GO annotations, the significant difference of each category for each group compared to the whole genome in TAIR8 was calculated with a poisson p-value for data in Figure 5C and Table S5. For the small number of genes in developmental process and transcription categories, Fisher's exact test was used for assessing the significance of data in Table 1.

### ChIP–PCR

To validate EMF1-bound genes identified by ChIP-chip, ChIP products from three independent biological samples were used to perform semi-quantitative PCR according to Moon et al., [33] on genes with different p-values. The PCR bands were scanned and measured by ImageJ program (http://imagej.nih.gov/ij/). The input signal for each gene was normalized to 100. The IP signal was calculated as % input. *PHE1*, which is not bound by EMF1, was used as negative control and its IP signal was subtracted from that of other genes and plotted on the graph. Primer sequences used for ChIP analysis are listed in Table S11.

### RT–PCR

Total RNA was extracted from WT, *emf1*, and *emf2* according to Moon et al., [33]. Semi-quantitative PCR was performed as described previously [33]. Primers used were as follows: EMF1; (AGGTGCTGCCAACGAGATTGAT and CTTTTGAGTTTGAATGCAGTCCAC), *UBQ*; ( *GATCTTTGCCGGAAAACAATTGGAGGATGGT* and *CGACTTGTCATTAGAAAGAAAGAGATAACAGG* ). RT-PCR of all samples and reference controls were performed in 3 independent replicates and repeated at least three times with similar results.

### Data deposition

Sequences are deposited in Gene Expression Omnibus (GEO) with accession number GSE34689.

## Supporting Information

Figure S1 EMF1 binding and H3K27me3 pattern in WT. (A) Genome browser view of Chromosome I with EMF1 binding and H3K27me3 in WT. Black box represents gene body. Y-axis represents log2-ratio of the IP/input signals. (B) EMF1-binding and H3K27me3 modification on flower organ-specific genes. 10 flower *MADS box* genes and *CRABS CLAW* (*CRC*) are EMF1-bound and trimethylated on H3K27 in WT seedlings. The H3K27me3 modification is reduced to varying degrees in the three mutants on these genes. *AG*: *AGAMOUS*; *PI*: *PISTILATA*; *AP1/2*: *APETALA1/3*; *SHP1/2*: *SHATTERPROOF1/2*; *SEP1/2/3*: *SEPALATA1/2/3*; *CAL*: *CAULIFLOWER*.
(PDF)

Figure S2 ChIP-PCR of EMF1-bound genes. Four genes with high (p<10^−20^) and five genes with low (10^−18^<p<10^−6^) enrichment of EMF1-3FLAG binding were randomly selected to confirm EMF1 binding by ChIP-PCR. *PHE1* is used as negative control. Gene ID and the p-value of EMF1 binding from the ChIP-chip data are shown in the Table. ChIP products from three independent biological samples were used to perform semi-quantitative PCR, using primer sequences located within 500 bp from the TSS (see Table S7 for primer sequences). Results are shown in the graph. Twenty eight to thirty five PCR cycles were performed for each gene. Three PCR experiments were performed with the cycles showing enrichment. Average IP from three experiments were expressed on the graph as % of corresponding input DNA. Error bars represent the standard deviations. Results were consistent among the 3 biological samples (see Materials and Methods).
(PDF)

Figure S3 EMF1-independent repression of *PHE1* and *PHE2*. EMF1 binding and H3K27me3 pattern on *PHE1* and *PHE2* chromatin on WT and 3 mutants, and RNA expression change of *PHE1* and *PHE2* from WT in the three mutants. *PHE1/2*: *PHERES1/2*.
(PDF)

Figure S4 Expression change of EMF1_no_K27 genes. (A) Expression change of 2303 EMF1_no_K27 genes in three mutants. All data based on NimbleGen microarray analysis. (B) Coordinated regulation of EMF1-bound photosynthesis genes by EMF1 and PRC2. *At1g03130: PHOTOSYSTEM I SUBUNIT D-2*, *At1g15820:LIGHT HARVESTING COMPLEX OF PHOTOSYSTEM II SUBUNIT6 (LHCB6), At1g31330:PHOTOSYSTEM I SUBUNIT F, At2g34430: LIGHT-HARVESTING CHLOROPHYLL PROTEIN COMPLEX II SUBUNIT B1, At3g08940: LHCB4.2, At5g01530: LCHB4.1*.
(PDF)

Table S1 EMF1 binding peaks on the 5 Arabidopsis chromosomes.
(XLS)

Table S2 Lists of H3K27me3 (K27), EMF1-bound, EMF1_K27, Group I, Group II, and EMF1_no_K27 genes.
(XLS)

Table S3 Overrepresented GO categories (molecular function, developmental, hormone response and stress) in EMF1_K27 genes.
(XLS)

Table S4 A subset of H3K27trimethylated genes are EMF1-bound.
(XLS)

Table S5 Percent representation of 9 functional GO categories of EMF1_K27 genes shown in Figure 5C.
(XLS)

Table S6 Select EMF1_K27 genes coordinately regulated by EMF1 and PRC2.
(XLS)

Table S7 Primer sequences used in ChIP-PCR experiments.
(XLS)

## References

[1] Lee TI, Jenner RG, Boyer LA, Guenther MG, Levine SS (2006). Control of developmental regulators by Polycomb in human embryonic stem cells.. Cell.

[2] Schwartz YB, Kahn TG, Nix DA, Li XY, Bourgon R (2006). Genome-wide analysis of Polycomb targets in Drosophila melanogaster.. Nat Genet.

[3] Klymenko T, Papp B, Fischle W, Kocher T, Schelder M (2006). A Polycomb group protein complex with sequence-specific DNA-binding and selective methyl-lysine-binding activities.. Genes Dev.

[4] Cao R, Wang L, Wang H, Xia L, Erdjument-Bromage H (2002). Role of histone H3 lysine 27 methylation in Polycomb-group silencing.. Science.

[5] Cao R, Zhang Y (2004). The functions of E(Z)/EZH2-mediated methylation of lysine 27 in histone H3.. Curr Opin Genet Dev.

[6] Czermin B, Melfi R, McCabe D, Seitz V, Imhof A (2002). Drosophila enhancer of Zeste/ESC complexes have a histone H3 methyltransferase activity that marks chromosomal Polycomb sites.. Cell.

[7] Kuzmichev A, Nishioka K, Erdjument-Bromage H, Tempst P, Reinberg D (2002). Histone methyltransferase activity associated with a human multiprotein complex containing the Enhancer of Zeste protein.. Genes Dev.

[8] Muller R, Goodrich J (2011). Sweet memories: epigenetic control in flowering.. F1000 Biol Rep.

[9] Dellino GI, Schwartz YB, Farkas G, McCabe D, Elgin SC (2004). Polycomb silencing blocks transcription initiation.. Mol Cell.

[10] King IF, Emmons RB, Francis NJ, Wild B, Muller J (2005). Analysis of a polycomb group protein defines regions that link repressive activity on nucleosomal templates to in vivo function.. Mol Cell Biol.

[11] Francis NJ, Kingston RE, Woodcock CL (2004). Chromatin compaction by a polycomb group protein complex.. Science.

[12] Ku M, Koche RP, Rheinbay E, Mendenhall EM, Endoh M (2008). Genomewide analysis of PRC1 and PRC2 occupancy identifies two classes of bivalent domains.. PLoS Genet.

[13] Majewski IJ, Ritchie ME, Phipson B, Corbin J, Pakusch M (2011). Opposing roles of polycomb repressive complexes in hematopoietic stem and progenitor cells.. Blood.

[14] Ringrose L, Paro R (2004). Epigenetic regulation of cellular memory by the Polycomb and Trithorax group proteins.. Annu Rev Genet.

[15] Chanvivattana Y, Bishopp A, Schubert D, Stock C, Moon YH (2004). Interaction of Polycomb-group proteins controlling flowering in Arabidopsis.. Development.

[16] Schubert D, Clarenz O, Goodrich J (2005). Epigenetic control of plant development by Polycomb-group proteins.. Curr Opin Plant Biol.

[17] Wood CC, Robertson M, Tanner G, Peacock WJ, Dennis ES (2006). The Arabidopsis thaliana vernalization response requires a polycomb-like protein complex that also includes VERNALIZATION INSENSITIVE 3.. Proc Natl Acad Sci U S A.

[18] Kinoshita T, Harada JJ, Goldberg RB, Fischer RL (2001). Polycomb repression of flowering during early plant development.. Proc Natl Acad Sci U S A.

[19] Hennig L, Taranto P, Walser M, Schonrock N, Gruissem W (2003). Arabidopsis MSI1 is required for epigenetic maintenance of reproductive development.. Development.

[20] Yoshida N, Yanai Y, Chen L, Kato Y, Hiratsuka J (2001). EMBRYONIC FLOWER2, a novel polycomb group protein homolog, mediates shoot development and flowering in Arabidopsis.. Plant Cell.

[21] Gendall AR, Levy YY, Wilson A, Dean C (2001). The VERNALIZATION 2 gene mediates the epigenetic regulation of vernalization in Arabidopsis.. Cell.

[22] Grossniklaus U, Vielle-Calzada JP, Hoeppner MA, Gagliano WB (1998). Maternal control of embryogenesis by MEDEA, a polycomb group gene in Arabidopsis.. Science.

[23] Goodrich J, Puangsomlee P, Martin M, Long D, Meyerowitz EM (1997). A Polycomb-group gene regulates homeotic gene expression in Arabidopsis.. Nature.

[24] Schatlowski N, Creasey K, Goodrich J, Schubert D (2008). Keeping plants in shape: polycomb-group genes and histone methylation.. Semin Cell Dev Biol.

[25] Calonje M, Sung ZR (2006). Complexity beneath the silence.. Curr Opin Plant Biol.

[26] Hennig L, Derkacheva M (2009). Diversity of Polycomb group complexes in plants: same rules, different players?. Trends Genet.

[27] Yang CH, Chen LJ, Sung ZR (1995). Genetic regulation of shoot development in Arabidopsis: role of the EMF genes.. Dev Biol.

[28] Aubert D, Chen L, Moon YH, Martin D, Castle LA (2001). EMF1, a novel protein involved in the control of shoot architecture and flowering in Arabidopsis.. Plant Cell.

[29] Sung ZR, Belachew A, Shunong B, Bertrand-Garcia R (1992). EMF, an Arabidopsis Gene Required for Vegetative Shoot Development.. Science.

[30] Bouyer D, Roudier F, Heese M, Andersen ED, Gey D (2011). Polycomb repressive complex 2 controls the embryo-to-seedling phase transition.. PLoS Genet.

[31] Sanchez R, Kim MY, Calonje M, Moon YH, Sung ZR (2009). Temporal and spatial requirement of EMF1 activity for Arabidopsis vegetative and reproductive development.. Mol Plant.

[32] Chen L, Cheng JC, Castle L, Sung ZR (1997). EMF genes regulate Arabidopsis inflorescence development.. Plant Cell.

[33] Moon YH, Chen L, Pan RL, Chang HS, Zhu T (2003). EMF genes maintain vegetative development by repressing the flower program in Arabidopsis.. Plant Cell.

[34] Kim SY, Zhu T, Sung ZR (2010). Epigenetic regulation of gene programs by EMF1 and EMF2 in Arabidopsis.. Plant Physiol.

[35] Alexandre C, Moller-Steinbach Y, Schonrock N, Gruissem W, Hennig L (2009). Arabidopsis MSI1 is required for negative regulation of the response to drought stress.. Mol Plant.

[36] Calonje M, Sanchez R, Chen L, Sung ZR (2008). EMBRYONIC FLOWER1 participates in polycomb group-mediated AG gene silencing in Arabidopsis.. Plant Cell.

[37] Bratzel F, Lopez-Torrejon G, Koch M, Del Pozo JC, Calonje M (2010). Keeping cell identity in Arabidopsis requires PRC1 RING-finger homologs that catalyze H2A monoubiquitination.. Curr Biol.

[38] Cao R, Tsukada Y, Zhang Y (2005). Role of Bmi-1 and Ring1A in H2A ubiquitylation and Hox gene silencing.. Mol Cell.

[39] Sanchez-Pulido L, Devos D, Sung ZR, Calonje M (2008). RAWUL: a new ubiquitin-like domain in PRC1 ring finger proteins that unveils putative plant and worm PRC1 orthologs.. BMC Genomics.

[40] Xu L, Shen WH (2008). Polycomb silencing of KNOX genes confines shoot stem cell niches in Arabidopsis.. Curr Biol.

[41] Turck F, Roudier F, Farrona S, Martin-Magniette ML, Guillaume E (2007). Arabidopsis TFL2/LHP1 specifically associates with genes marked by trimethylation of histone H3 lysine 27.. PLoS Genet.

[42] Zhang X, Clarenz O, Cokus S, Bernatavichute YV, Pellegrini M (2007). Whole-genome analysis of histone H3 lysine 27 trimethylation in Arabidopsis.. PLoS Biol.

[43] Park HY, Lee SY, Seok HY, Kim SH, Sung ZR (2011). EMF1 Interacts with EIP1, EIP6 or EIP9 Involved in the Regulation of Flowering Time in Arabidopsis.. Plant Cell Physiol.

[44] To A, Valon C, Savino G, Guilleminot J, Devic M (2006). A network of local and redundant gene regulation governs Arabidopsis seed maturation.. Plant Cell.

[45] Griffith ME, da Silva Conceicao A, Smyth DR (1999). PETAL LOSS gene regulates initiation and orientation of second whorl organs in the Arabidopsis flower.. Development.

[46] Brewer PB, Howles PA, Dorian K, Griffith ME, Ishida T (2004). PETAL LOSS, a trihelix transcription factor gene, regulates perianth architecture in the Arabidopsis flower.. Development.

[47] Alvarez-Buylla ER, Pelaz S, Liljegren SJ, Gold SE, Burgeff C (2000). An ancestral MADS-box gene duplication occurred before the divergence of plants and animals.. Proc Natl Acad Sci U S A.

[48] Bemer M, Heijmans K, Airoldi C, Davies B, Angenent GC (2010). An atlas of type I MADS box gene expression during female gametophyte and seed development in Arabidopsis.. Plant Physiol.

[49] Masiero S, Colombo L, Grini PE, Schnittger A, Kater MM (2011). The emerging importance of type I MADS box transcription factors for plant reproduction.. Plant Cell.

[50] Aida M, Tasaka M (2006). Genetic control of shoot organ boundaries.. Curr Opin Plant Biol.

[51] Aida M, Ishida T, Fukaki H, Fujisawa H, Tasaka M (1997). Genes involved in organ separation in Arabidopsis: an analysis of the cup-shaped cotyledon mutant.. Plant Cell.

[52] Ha CM, Jun JH, Fletcher JC (2010). Control of Arabidopsis leaf morphogenesis through regulation of the YABBY and KNOX families of transcription factors.. Genetics.

[53] Kieffer M, Master V, Waites R, Davies B (2011). TCP14 and TCP15 affect internode length and leaf shape in Arabidopsis.. Plant J.

[54] Lafos M, Kroll P, Hohenstatt ML, Thorpe FL, Clarenz O (2011). Dynamic regulation of H3K27 trimethylation during Arabidopsis differentiation.. PLoS Genet.

[55] Rubio-Somoza I, Weigel D (2011). MicroRNA networks and developmental plasticity in plants.. Trends Plant Sci.

[56] Wu G, Park MY, Conway SR, Wang JW, Weigel D (2009). The sequential action of miR156 and miR172 regulates developmental timing in Arabidopsis.. Cell.

[57] Mallory A, Vaucheret H (2010). Form, function, and regulation of ARGONAUTE proteins.. Plant Cell.

[58] Ha CM, Jun JH, Nam HG, Fletcher JC (2004). BLADE-ON-PETIOLE1 encodes a BTB/POZ domain protein required for leaf morphogenesis in Arabidopsis thaliana.. Plant Cell Physiol.

[59] Karim MR, Hirota A, Kwiatkowska D, Tasaka M, Aida M (2009). A role for Arabidopsis PUCHI in floral meristem identity and bract suppression.. Plant Cell.

[60] Chini A, Fonseca S, Fernandez G, Adie B, Chico JM (2007). The JAZ family of repressors is the missing link in jasmonate signalling.. Nature.

[61] Jun J, Fiume E, Roeder AH, Meng L, Sharma VK (2010). Comprehensive analysis of CLE polypeptide signaling gene expression and overexpression activity in Arabidopsis.. Plant Physiol.

[62] Strabala TJ, O'Donnell PJ, Smit AM, Ampomah-Dwamena C, Martin EJ (2006). Gain-of-function phenotypes of many CLAVATA3/ESR genes, including four new family members, correlate with tandem variations in the conserved CLAVATA3/ESR domain.. Plant Physiol.

[63] Bond DM, Dennis ES, Finnegan EJ (2011). The low temperature response pathways for cold acclimation and vernalization are independent.. Plant Cell Environ.

[64] Carles CC, Fletcher JC (2010). Missing links between histones and RNA Pol II arising from SAND?. Epigenetics.

[65] Kotake T, Takada S, Nakahigashi K, Ohto M, Goto K (2003). Arabidopsis TERMINAL FLOWER 2 gene encodes a heterochromatin protein 1 homolog and represses both FLOWERING LOCUS T to regulate flowering time and several floral homeotic genes.. Plant Cell Physiol.

[66] Bratzel F, Yang C, Angelova A, Lopez-Torrejon G, Koch M (2011). Regulation of the New Arabidopsis Imprinted Gene AtBMI1C Requires the Interplay of Different Epigenetic Mechanisms.. Mol Plant.

[67] Simon JAKR (2009). Mechanism of polycomb gene silencing: knowns and unknowns.. Nat Rev Mol Cell Biol.

[68] Schwartz YB, Kahn TG, Stenberg P, Ohno K, Bourgon R (2010). Alternative epigenetic chromatin states of polycomb target genes.. PLoS Genet.

[69] Schoeftner S, Sengupta AK, Kubicek S, Mechtler K, Spahn L (2006). Recruitment of PRC1 function at the initiation of X inactivation independent of PRC2 and silencing.. Embo J.

[70] Chen D, Molitor A, Liu C, Shen WH (2010). The Arabidopsis PRC1-like ring-finger proteins are necessary for repression of embryonic traits during vegetative growth.. Cell Res.

[71] Kallin EM, Cao R, Jothi R, Xia K, Cui K (2009). Genome-wide uH2A localization analysis highlights Bmi1-dependent deposition of the mark at repressed genes.. PLoS Genet.

[72] Chen LJ, Diao ZY, Specht C, Sung ZR (2009). Molecular evolution of VEF-domain-containing PcG genes in plants.. Mol Plant.

[73] Luo M, Platten D, Chaudhury A, Peacock WJ, Dennis ES (2009). Expression, imprinting, and evolution of rice homologs of the polycomb group genes.. Mol Plant.

[74] Weinhofer I, Hehenberger E, Roszak P, Hennig L, Kohler C (2010). H3K27me3 profiling of the endosperm implies exclusion of polycomb group protein targeting by DNA methylation.. PLoS Genet.

[75] Carles CC, Fletcher JC (2009). The SAND domain protein ULTRAPETALA1 acts as a trithorax group factor to regulate cell fate in plants.. Genes Dev.

[76] Murashige T, Skoog F (1962). A revised medium for rapid growth and bioassays with tocacco tissue cultures.. Physiological Plant.

[77] Bowler C, Benvenuto G, Laflamme P, Molino D, Probst AV (2004). Chromatin techniques for plant cells.. Plant J.

[78] Zilberman D, Gehring M, Tran RK, Ballinger T, Henikoff S (2007). Genome-wide analysis of Arabidopsis thaliana DNA methylation uncovers an interdependence between methylation and transcription.. Nat Genet.

[79] Zilberman D, Coleman-Derr D, Ballinger T, Henikoff S (2008). Histone H2A.Z and DNA methylation are mutually antagonistic chromatin marks.. Nature.

[80] Buck MJ, Nobel AB, Lieb JD (2005). ChIPOTle: a user-friendly tool for the analysis of ChIP-chip data.. Genome Biol.

[81] Berardini TZ, Mundodi S, Reiser L, Huala E, Garcia-Hernandez M (2004). Functional annotation of the Arabidopsis genome using controlled vocabularies.. Plant Physiol.

