# Supplementary figures and images for: EMF1 and PRC2 Cooperate to Repress Key Regulators of Arabidopsis Development
Source: PLoS Genet. 2012 Mar 22;8(3):e1002512. doi: 10.1371/journal.pgen.1002512 (PMC3310727; doi:10.1371/journal.pgen.1002512)

Figure S1

A

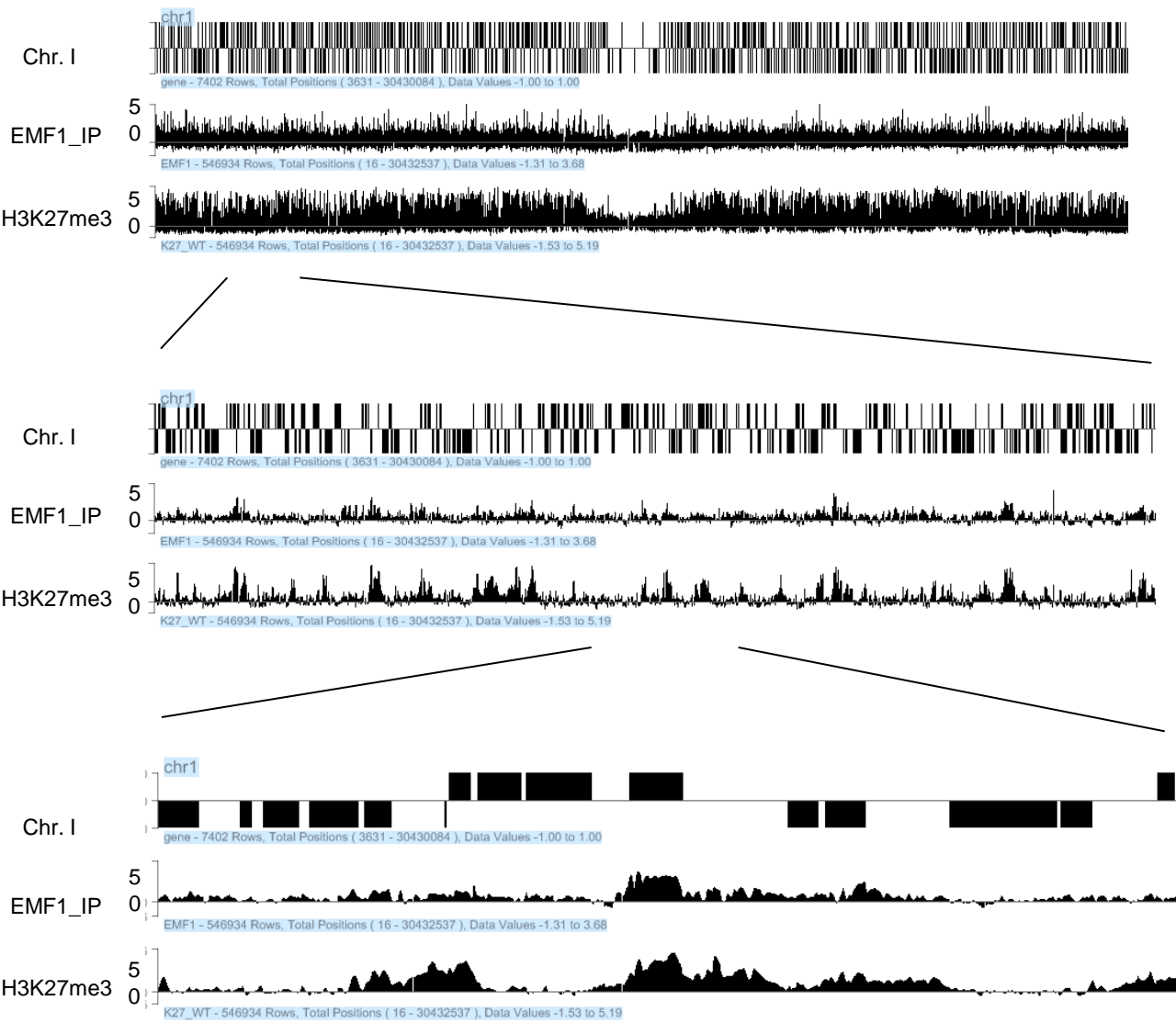

B

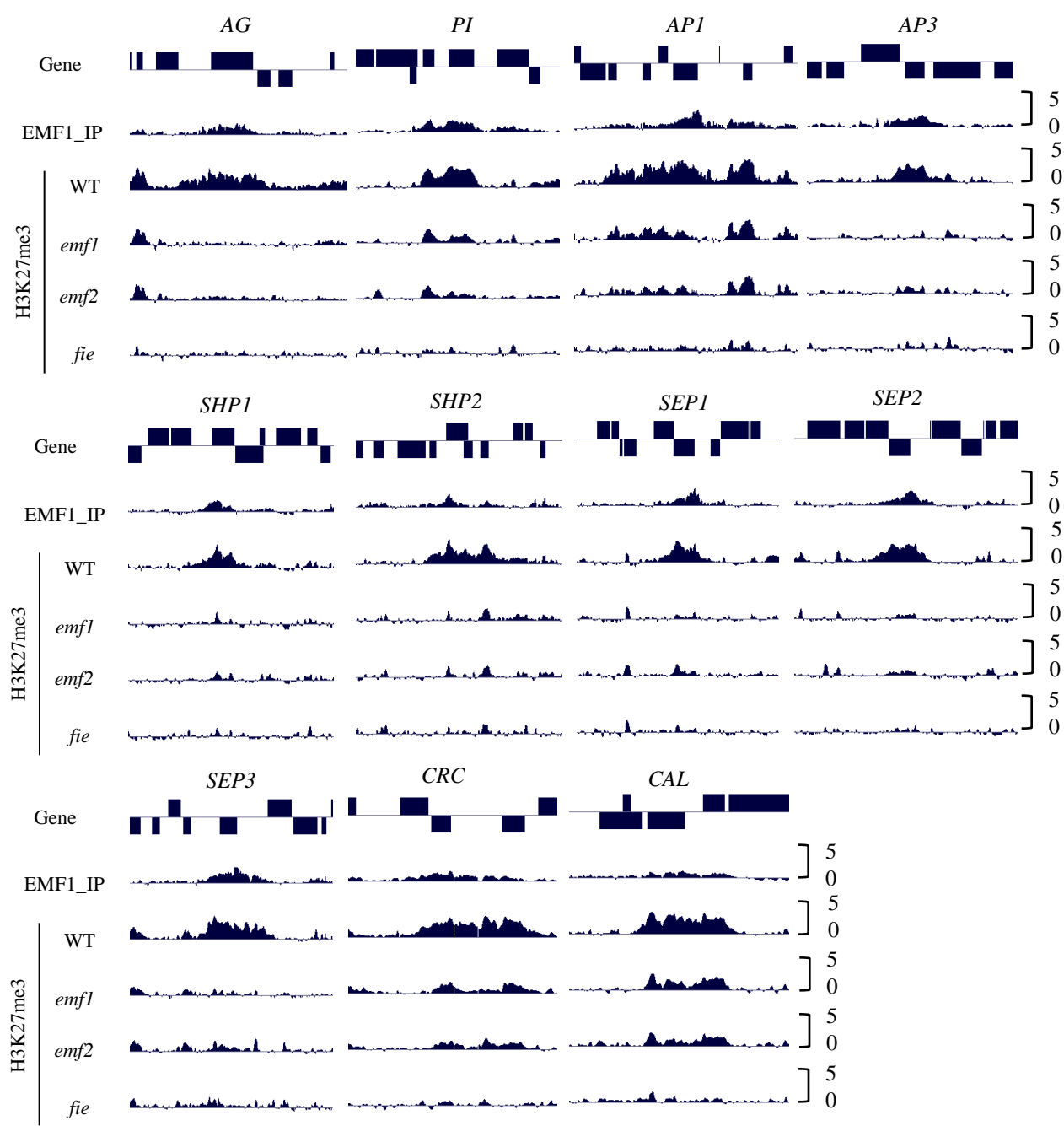

Supplement: Figure S1 — EMF1 binding and H3K27me3 pattern in WT. (A) Genome browser view of Chromosome I with EMF1 binding and H3K27me3 in WT. Black box represents gene body. Y-axis represents log2-ratio of the IP/input signals. (B) EMF1-binding and H3K27me3 modification on flower organ-specific genes. 10 flower MADS box genes and CRABS CLAW (CRC) are EMF1-bound and trimethylated on H3K27 in WT seedlings. The H3K27me3 modification is reduced to varying degrees in the three mutants on these genes. AG: AGAMOUS; PI: PISTILATA; AP1/2: APETALA1/3; SHP1/2: SHATTERPROOF1/2; SEP1/2/3: SEPALATA1/2/3; CAL: CAULIFLOWER. (PDF) [file pgen.1002512.s001.pdf]

Figure S3

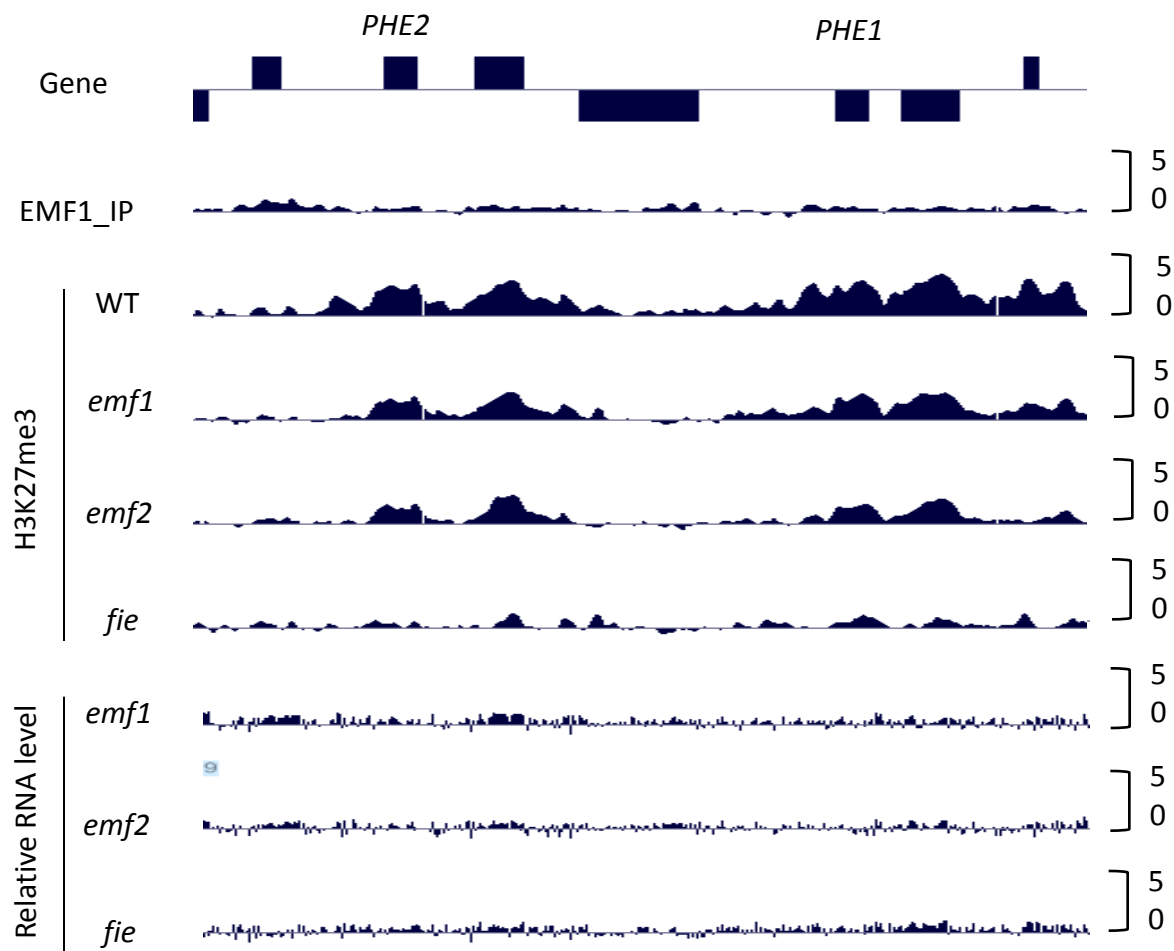

Supplement: Figure S3 — EMF1-independent repression of PHE1 and PHE2. EMF1 binding and H3K27me3 pattern on PHE1 and PHE2 chromatin on WT and 3 mutants, and RNA expression change of PHE1 and PHE2 from WT in the three mutants. PHE1/2: PHERES1/2. (PDF) [file pgen.1002512.s003.pdf]

Figure S4

A

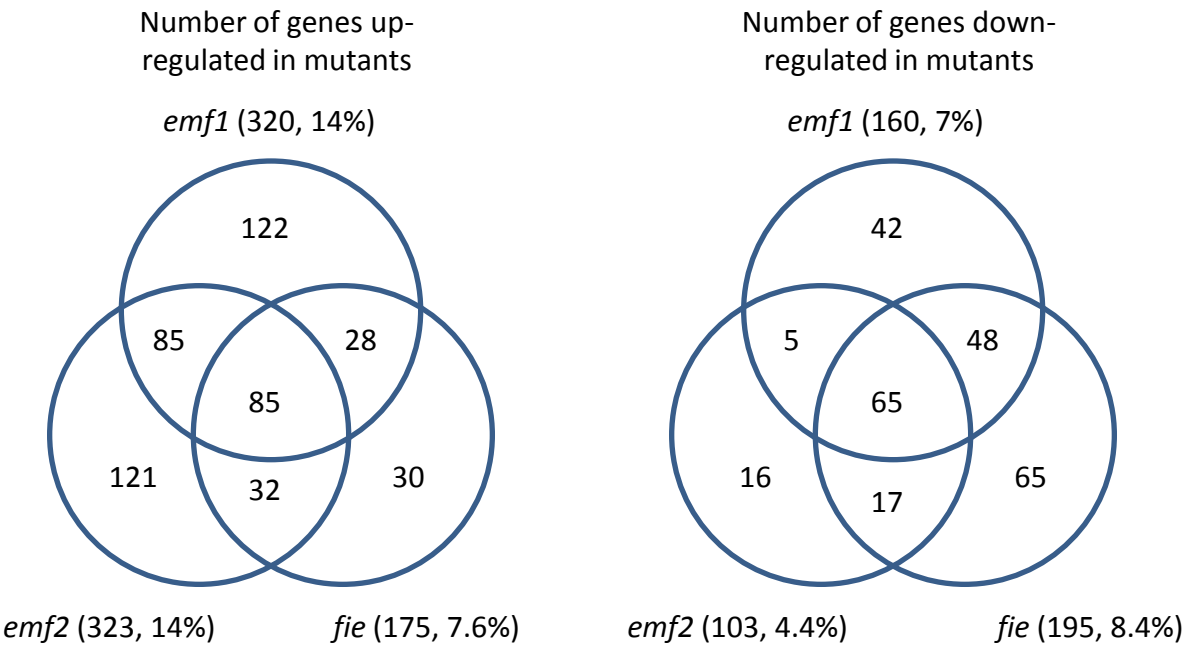

B

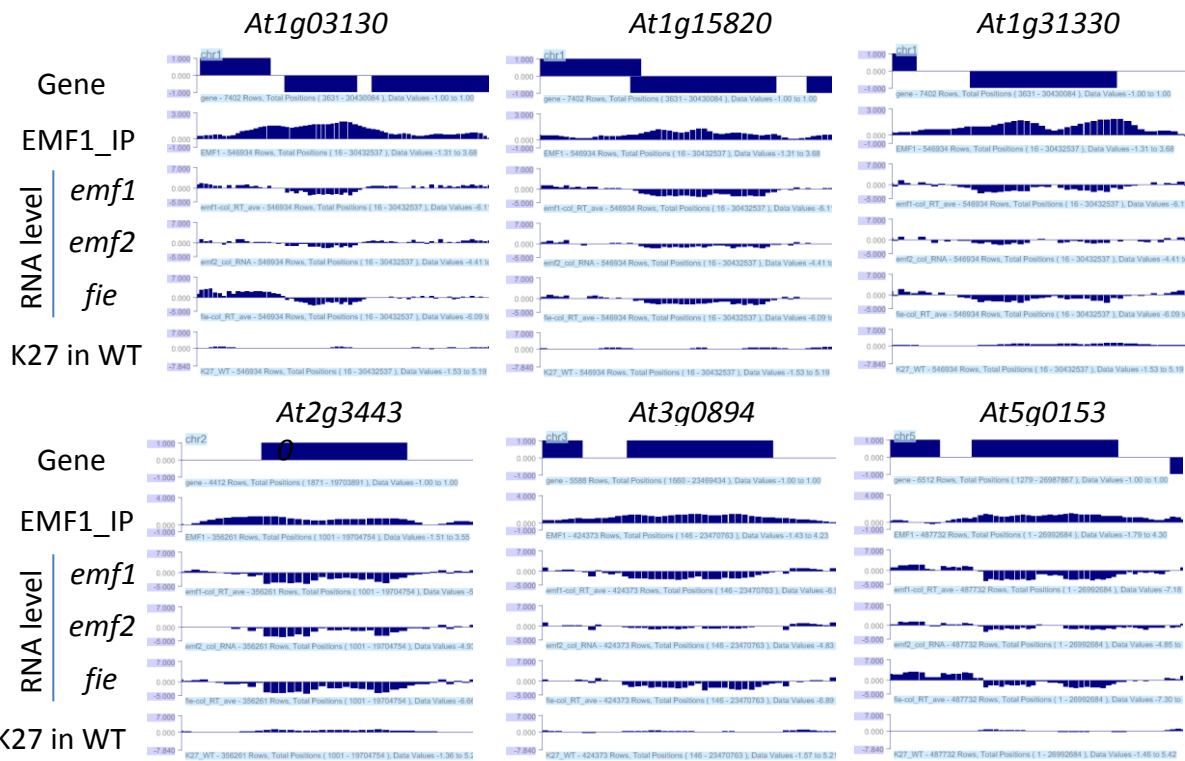

Supplement: Figure S4 — Expression change of EMF1_no_K27 genes. (A) Expression change of 2303 EMF1_no_K27 genes in three mutants. All data based on NimbleGen microarray analysis. (B) Coordinated regulation of EMF1-bound photosynthesis genes by EMF1 and PRC2. At1g03130: PHOTOSYSTEM I SUBUNIT D-2, At1g15820:LIGHT HARVESTING COMPLEX OF PHOTOSYSTEM II SUBUNIT6 (LHCB6), At1g31330:PHOTOSYSTEM I SUBUNIT F, At2g34430: LIGHT-HARVESTING CHLOROPHYLL PROTEIN COMPLEX II SUBUNIT B1, At3g08940: LHCB4.2, At5g01530: LCHB4.1. (PDF) [file pgen.1002512.s004.pdf]
