# Supplementary material for: EMF1 and PRC2 Cooperate to Repress Key Regulators of Arabidopsis Development
Source: PLoS Genet. 2012 Mar 22;8(3):e1002512. doi: 10.1371/journal.pgen.1002512 (PMC3310727; doi:10.1371/journal.pgen.1002512)

Figure S2

Gene ID	Gene Symbol	EMF1_IP (p- value)	Data source	Gene ID	Gene Symbol	EMF1_IP (p- value)	Data source
AT1g65330	PHE1	$10^{-6} < p$	Supplemental Figure S2 & Kim et al., 2010	AT4g18390	TCP2	$p < 10^{-20}$	Supplemental Figure S2
AT3g23050	IAA7	$10^{-10} < p < 10^{-6}$	Supplemental Figure S2	At1g62500	LTP	$p < 10^{-20}$	Supplemental Figure S2
AT5g07200	GA20OX3	$10^{-10} < p < 10^{-6}$	Supplemental Figure S2	At4g18960	AG	$p < 10^{-20}$	Calonje et al., 2008
At1g80130	TPR-like	$10^{-15} < p < 10^{-10}$	Supplemental Figure S2	At3g54340	AP3	$p < 10^{-20}$	Calonje et al., 2008
At4g08940	Ubiquitin carboxyl terminal hydrolase	$10^{-18} < p < 10^{-15}$	Supplemental Figure S2	At5g20240	PI	$p < 10^{-20}$	Caonje et al., 2008 & Kim et al., 2010
AT4G27140	SESA1	$10^{-10} < p < 10^{-6}$	Supplemental Figure S2	At3g24650	ABI3	$p < 10^{-20}$	Kim et al., 2010
At2g40000	HSPRO2	$p < 10^{-20}$	Supplemental Figure S2	At2g02450	LOV1	$p < 10^{-20}$	Kim et al., 2010
AT1g23380	KNAT6	$p < 10^{-20}$	Supplemental Figure S2	At5g10140	FLC	$p < 10^{-20}$	Kim et al., 2010

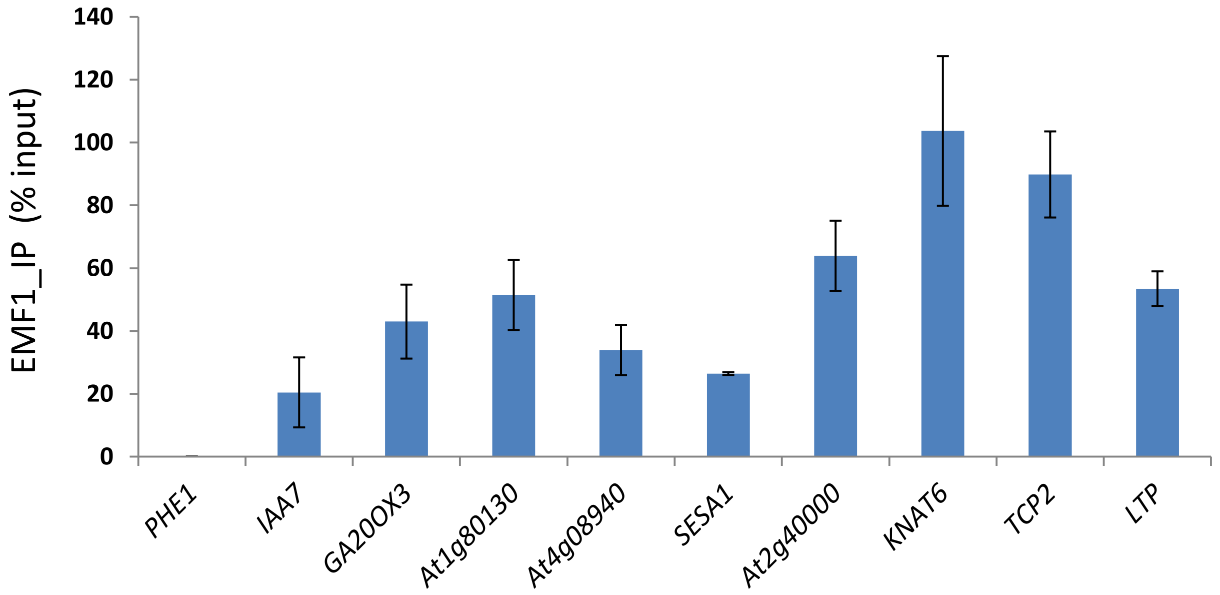

Supplement: Figure S2 — ChIP-PCR of EMF1-bound genes. Four genes with high (p<10−20) and five genes with low (10−18<p<10−6) enrichment of EMF1-3FLAG binding were randomly selected to confirm EMF1 binding by ChIP-PCR. PHE1 is used as negative control. Gene ID and the p-value of EMF1 binding from the ChIP-chip data are shown in the Table. ChIP products from three independent biological samples were used to perform semi-quantitative PCR, using primer sequences located within 500 bp from the TSS (see Table S7 for primer sequences). Results are shown in the graph. Twenty eight to thirty five PCR cycles were performed for each gene. Three PCR experiments were performed with the cycles showing enrichment. Average IP from three experiments were expressed on the graph as % of corresponding input DNA. Error bars represent the standard deviations. Results were consistent among the 3 biological samples (see Materials and Methods). (PDF) [file pgen.1002512.s002.pdf]
